# The primary familial brain calcification-associated protein MYORG is an α-galactosidase with restricted substrate specificity

**DOI:** 10.1371/journal.pbio.3001764

**Published:** 2022-09-21

**Authors:** Richard W. Meek, Jacob Brockerman, Osei B. Fordwour, Wesley F. Zandberg, Gideon J. Davies, David J. Vocadlo

**Affiliations:** 1 Department of Chemistry. University of York, York, United Kingdom; 2 Department of Molecular Biology and Biochemistry, Simon Fraser University, Burnaby, British Columbia, Canada; 3 Department of Chemistry, Simon Fraser University, Burnaby, British Columbia, Canada; 4 Department of Chemistry, Irving K. Barber Faculty of Science, University of British Columbia, Kelowna, British Columbia, Canada; Georgia Institute of Technology, UNITED STATES

## Abstract

Primary familial brain calcification (PFBC) is characterised by abnormal deposits of calcium phosphate within various regions of the brain that are associated with severe cognitive impairments, psychiatric conditions, and movement disorders. Recent studies in diverse populations have shown a link between mutations in myogenesis-regulating glycosidase (MYORG) and the development of this disease. MYORG is a member of glycoside hydrolase (GH) family 31 (GH31) and, like the other mammalian GH31 enzyme α-glucosidase II, this enzyme is found in the lumen of the endoplasmic reticulum (ER). Though presumed to act as an α-glucosidase due to its localization and sequence relatedness to α-glucosidase II, MYORG has never been shown to exhibit catalytic activity. Here, we show that MYORG is an α-galactosidase and present the high-resolution crystal structure of MYORG in complex with substrate and inhibitor. Using these structures, we map detrimental mutations that are associated with MYORG-associated brain calcification and define how these mutations may drive disease progression through loss of enzymatic activity. Finally, we also detail the thermal stabilisation of MYORG afforded by a clinically approved small molecule ligand, opening the possibility of using pharmacological chaperones to enhance the activity of mutant forms of MYORG.

## Abstract 2

MYORG is an enzyme genetically linked to primary familial brain calcification that has historically been presumed to act as an α-glucosidase. This study describes the crystal structure of dimeric MYORG and, surprisingly, reveals it to be an α-galactosidase with restricted specificity.

## Introduction

Primary familial brain calcification (PFBC), commonly referred to as Fahr’s syndrome, is a set of rare genetic disorders associated with abnormal bilateral deposits of calcium phosphate within various regions of the brain [1]. Significant calcification is associated with cognitive impairments, psychiatric conditions, and movement disorders [1]. PFBC was generally considered an autosomal dominant disorder caused by genetic abnormalities in just 4 genes: *SLC20A2*, *XPR1*, *PDGFRB*, and *PDGFB* [2–5]. Recent studies, however, have linked development of PFBC to biallelic loss-of-function mutations in the genes *JAM2* and *MYORG* (**S1 Table**) [6,7]. While the function of proteins encoded by *SLC20A2*, *XPR1*, *PDGFRB*, *PDGFB*, and *JAM2* have previously been described, and have offered some insights into their roles in PFBC progression [8–10], the function and activity of the myogenesis-regulating glycosidase (MYORG) encoded by *MYORG* remain unknown. Given that no treatment options are available for patients with PFBC, there is a need to understand the function of these proteins with the goal of understanding the root causes of PFBC.

**Fig 1:**
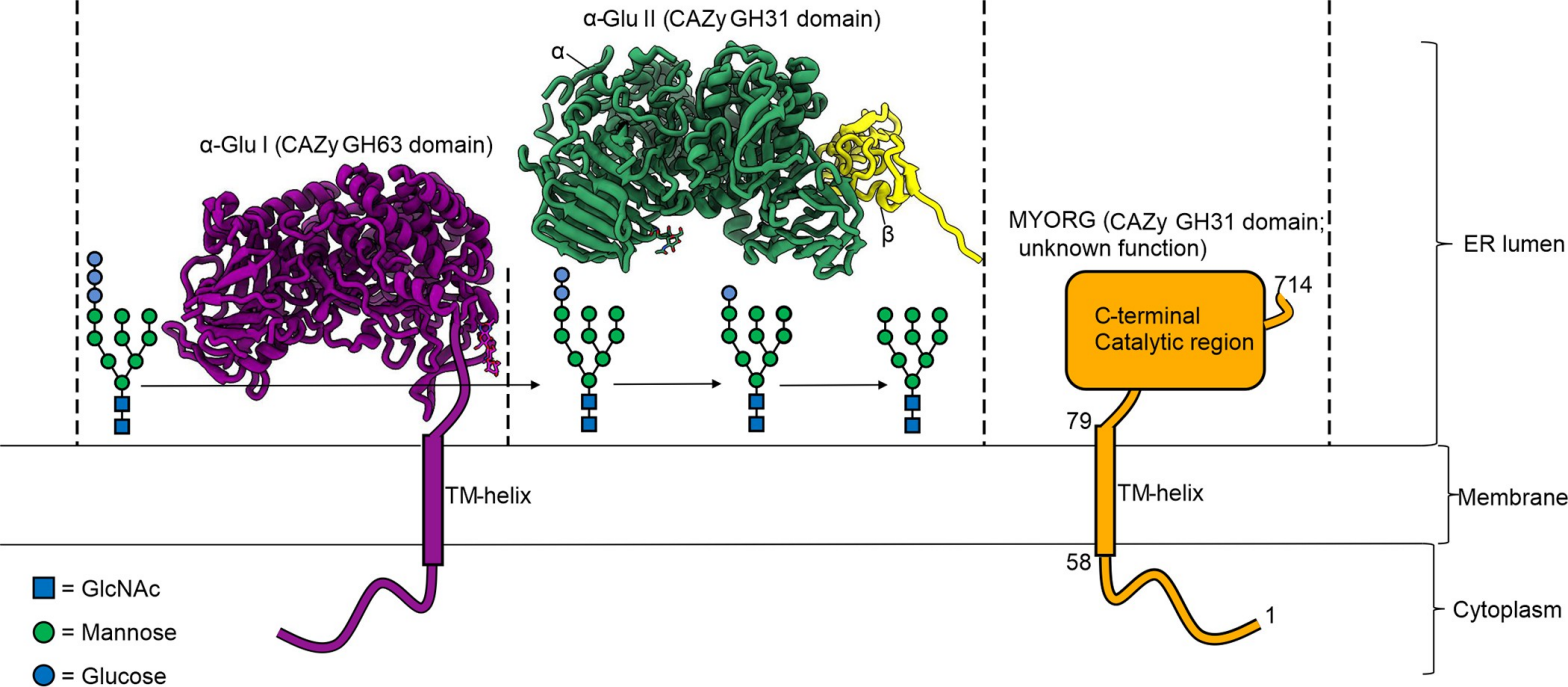
ER lumen α-glycosidases play roles in *N*-glycan processing. Crystal structures of *Mus musculus* α-glucosidase I (PDB: 5MHF) and II (PDB: 5F0E). The transfer of Glc_3_Man_9_GlcNAc_2_ onto nascent polypeptide chains initiates an ER localised quality control process wherein the terminal nonreducing α1-2-linked glucose and the 2 inner α1-3-linked glucose residues are hydrolysed by α-Glu I and α-Glu II, respectively. Retention in the ER of glycoproteins bearing the innermost α1-3-linked glucose by the chaperones calnexin and calreticulin coupled with re-attachment of α1-3-linked glucose to misfolded proteins by UDP-glucose:glycoprotein glucosyltransferase regulates protein quality control. The function of MYORG within the ER and relevance to glycoprotein processing is unknown. The symbols used for monosaccharides follow the recommendations of the CFG. CFG, Consortium for Functional Glycomics; ER, endoplasmic reticulum; MYORG, myogenesis-regulating glycosidase.

We were intrigued by MYORG, which is a type II transmembrane protein predicted to be comprised of a short, disordered nucleocytoplasmic N-terminal region, a single transmembrane helix, and a lumenal C-terminal catalytic region comprising a CAZy family glycoside hydrolase 31 (GH31) catalytic domain and 2 β-sheet domains (**Fig 1**) [11–13]. This GH31 domain has, for a glycan degrading enzyme, a distinctive localization within the early secretory pathway where most glycans typically start to be assembled. Within brain, MYORG is expressed in astrocytes, and in various cell lines, it has been shown to be distributed to the endoplasmic reticulum (ER) and nuclear envelope [6,11,14]. The lumenal orientation for the GH31 domain of MYORG has been demonstrated using protease digestion experiments [11]. This localization is similar to that seen for α-glucosidase I (α-Glu I; CAZy family GH63 domain) and the sequence-related GH31 α-glucosidase II (α-Glu II; CAZy family GH31 domain), which are 2 ER enzymes that play essential roles in protein quality control. Through their processing of terminal glucose residues from the *N*-glycans of newly synthesized glycoproteins, these 2 enzymes serve an essential role in regulating the engagement of glycoproteins within the calnexin/calreticulin cycle [15,16].

ER α-Glu I and α-Glu II act to cleave α-linked nonreducing glucose residues from a branch of the *N*-glycan, with α-Glu II using a double-displacement mechanism, which involves the transient formation of a glycosyl enzyme intermediate [17,18]. Based on the sequence similarity of MYORG to α-Glu II (25.2% seq ID; 53% sequence coverage) and conservation of the key catalytic residues, this enzyme has previously been assumed to be a catalytically active α-glucosidase [11,19]. Yet, attempts to demonstrate the activity of MYORG or identify any substrates for this enzyme have proved unsuccessful [11]. Understanding the substrate specificity of MYORG will help uncover the molecular mechanisms underlying development and progression of PFBC, as well as providing opportunities to design or repurpose existing drugs for treatment. Here, we demonstrate that MYORG, enigmatically, functions not as an α-glucosidase but rather an α-galactosidase and shows marked preference for specific disaccharide substrates. We use X-ray crystallography to obtain unliganded and both substrate and inhibitor bound structures of MYORG. We use these structures to pinpoint how disease-related mutations contribute to loss of function and downstream disease.

## Results

### MYORG is an active glycoside hydrolase that acts on α-galactosides

**Fig 2:**
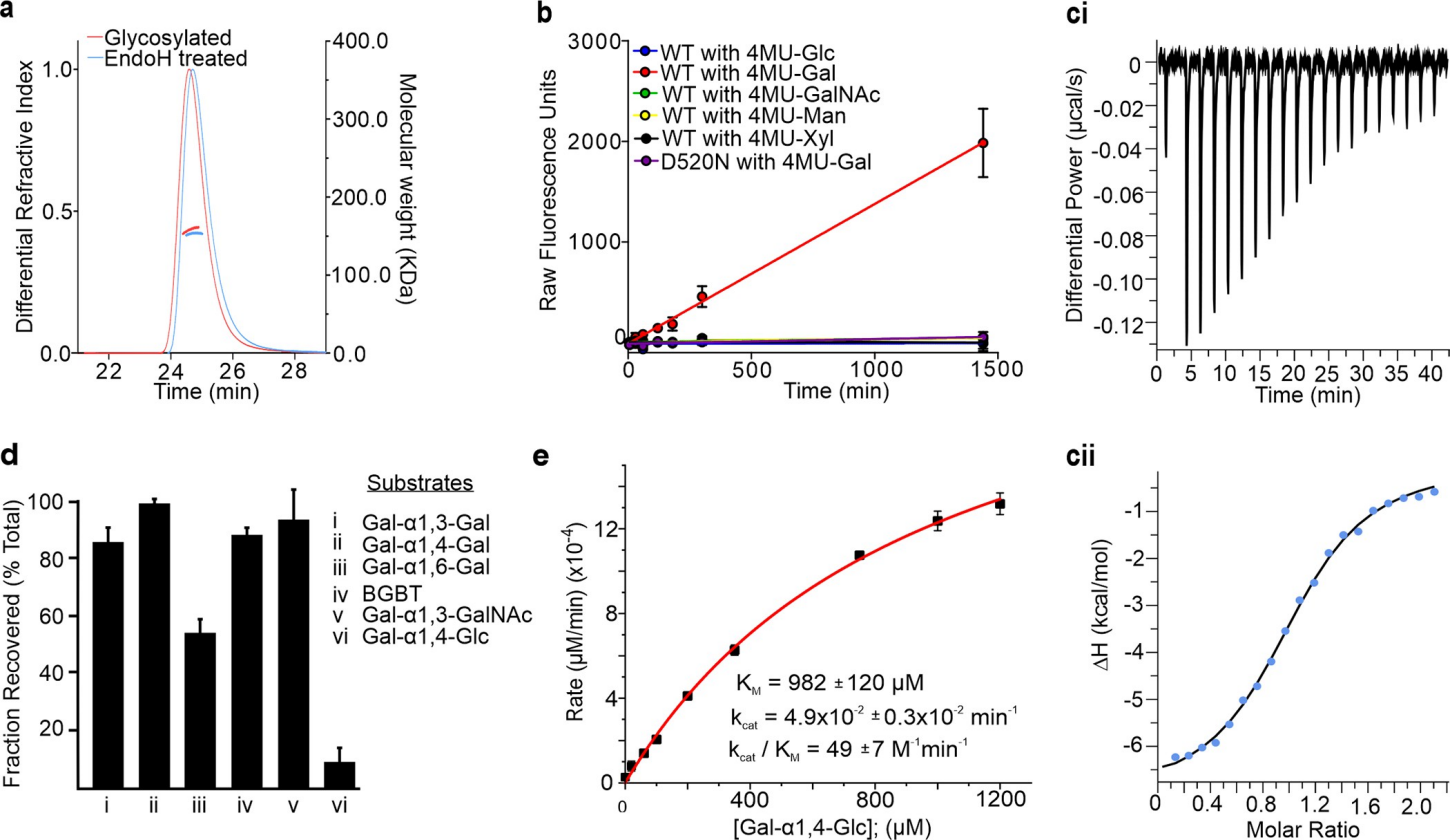
MYORG is a dimeric α-galactosidase that shows distinct substrate specificity. (**a**) SEC-MALLS traces of glycosylated and EndoH-treated MYORG. (**b**) Fluorescent activity assay of MYORG against 4MU-α-linked substrates. Data is mean from 3 technical replicates ± standard deviations. (**c**) Example isothermal titration calorimetry trace of DGJ binding to MYORG. (**ci**) Raw baseline subtracted injection profile of the ITC experiment. (**cii**) Titration curve with points in blue and fitted line in black. (**d**) Activity screening of MYORG against disaccharides. Experiment repeated twice with 3 technical repeats in each replicate. (**e**) Michaelis–Menten kinetics for processing of Gal-α1-4-Glc by MYORG. Data from 3 technical repeats. All raw data underlying graphs can be found in S1 Data. BGBT, blood group B trisaccharide; DGJ, deoxygalactonojirimycin; MYORG, myogenesis-regulating glycosidase.

Early efforts to recombinantly express MYORG in *Escherichia coli* proved unsuccessful in generating protein with detectable activity. Given that MYORG resides in the ER lumen and is therefore likely to be *N*-glycosylated, we reasoned that expression in a host system such as the eukaryote *Trichoplusia ni* could lead to active protein. Indeed, extensive glycosylation has previously been observed for MYORG derived from C2C12 cells, for which digestion by the *endo*-glycosidase EndoH, which cleaves *N*-glycans, leads to approximately 10 kDa reduction in molecular weight [11]. This sensitivity to EndoH digestion indicates the protein bears high mannose structures, which is consistent with its bearing *N*-glycans as expected of an ER localised protein. To focus on the function of the GH31 domain of MYORG and prevent membrane incorporation, we expressed residues 80–714 (MYORG_GH31_), trimming off the transmembrane domain (TMD) and the predicted N-terminal disordered region. We also introduced a His_6_ tag along with a TEV-protease cleavable N-terminal melittin signal sequence to drive secretion of the resulting protein product into the media. In this way, MYORG_GH31_ could be successfully purified from the media using metal-chelate affinity purification. To confirm glycosylation of MYORG, we treated the protein with EndoH and compared both the glycosylated and deglycosylated enzyme through size-exclusion chromatography multi-angle light scattering (SEC-MALLS). Complete digestion by EndoH was monitored through SDS-PAGE (**S1 Fig**). SEC-MALLS analysis suggested MYORG_GH31_ is 158 kDa in solution, which is close to the expected molecular weight of a dimer (154 kDa) (**Fig 2A**), whereas deglycosylated MYORG_GH31_ forms a 153 kDa complex, suggesting that glycosylation is not essential for dimerization and that MYORG_GH31_ is decorated with approximately 5 kDa of *N*-glycans. The relatively high level of *N*-glycosylation likely explains why previous efforts to express this protein in *E*.*coli* yielded apparently inactive protein.

With suitably folded and expressed MYORG_GH31_, we next analyzed whether this protein showed any activity against α-glucosides including standard chromogenic glucosides such as *para*-nitrophenyl α-D-glucopyranoside and the more sensitive fluorogenic 4-methylumbelliferyl α-D-glucopyranoside (4MU-Glc). However, we observed no activity against these substrates and therefore screened a panel of different 4-methylumbelliferyl (4MU) α-glycoside substrates including: α-D-mannopyranoside (4MU-Man), α-D-xylopyranoside (4MU-Xyl), α-D-galactopyranoside (4MU-Gal), and *N*-acetyl-α-D-galactosaminide (4MU-GalNAc) (**Fig 2B**). Substrate turnover was only observed for 4MU-Gal, suggesting that MYORG functions as an α-galactosidase. While we were unable to determine full kinetic parameters for 4MU-Gal, due to limited solubility of the substrate, we determined the value of the second-order rate constant k_cat_/K_M_ by linear regression of the Michaelis–Menten plot to be 434.8 ± 3.82 M^−1^ min^−1^ (**S2 Fig**). Though the enzyme shows apparent complete specificity for α-galactosides, this second-order rate constant for a highly activated substrate with an excellent activated phenolic leaving group is low. Though purified MYORG_GH31_ showed no bands other than MYORG, the low k_cat_/K_M_ value we observed left us concerned about the possibility that a low-level contaminant might be giving rise to this α-galactosidase activity. We therefore produced mutant protein in which the predicted general acid/base catalytic residue was mutated (Asp520Asn), which by analogy to other α-glycosidases from GH31 [20], should greatly reduce activity if MYORG_GH31_ is responsible for the observed catalysis. Reassuringly, this mutant MYORG_GH31_ failed to turn over 4MU-Gal indicating that MYORG is indeed an α-galactosidase (**Fig 2B**). Since MYORG is naturally glycosylated in cells, we only tested the recombinantly produced glycosylated form in kinetic assays.

We speculated that the low *k*_cat_/*K*_M_ value we observed for MYORG with 4MU-Gal might be explained by it having an unusual pH optimum for activity. Using 4MU-Gal we found, however, that MYORG has a relatively broad pH-activity profile with optimal activity at pH 6 (**S2 Fig**). Accordingly, the lack of any curvature in the Michaelis–Menten plot, coupled with the low *k*_cat_/*K*_M_ value we observed, suggested to us that the 4MU leaving group compromised substrate binding and resulted in what must be a very high *K*_M_ value for this substrate. To further probe the specificity of MYORG, we reasoned that, as a GH31 α-galactosidase, MYORG should bind and be inhibited by deoxynojirimycin analogues, given that deoxynojirimycin itself binds to GH31 *Mus* musculus (*Mm*) α-Glu II with a *IC*_50_ value of 11.4 μM [21]. We therefore reasoned that deoxygalactonojirimycin (DGJ), which is clinically approved as the pharmacological chaperone Migalastat that helps mutant forms of the GH27 α-galactosidase GalA that are found in Fabry disease patients to fold more efficiently and traffic to the lysosome [22,23], should bind similarly well to MYORG. Consistent with this view, we observed inhibition of MYORG activity towards 4MU-Gal in the presence of DGJ (**S2 Fig**). We then used isothermal titration calorimetry to determine a *K*_D_ value of 1.33 ± 0.45 μM (S3 Table) for binding of DGJ to MYORG_GH31_ (**Fig 2C**). Notably, the observation that MYORG_GH31_ binds tightly to DGJ lends further support for MYORG functioning as an α-galactosidase and also suggests the 4MU leaving group may hinder substrate binding to this enzyme.

Aryl glycosides are sometimes poor substrate for glycoside hydrolases because there exists a +1 binding site on the reducing side of the scissile bond that has a distinct preference for a carbohydrate residue [24]. We therefore set out to screen various disaccharide substrates to discern the substrate preference of MYORG. We elected to examine all of the α-galactose containing disaccharide structures that are known to exist in humans including: Gal-α1,3-Gal, Gal-α1,3-GalNAc, and Gal-α1,4-Gal. Surprisingly, using sensitive capillary electrophoresis analyses, we found that none of these substrates were significantly processed by MYORG (**Fig 2D**). We therefore turned to screening of available disaccharides containing a nonreducing α-galactoside (**Fig 2D**) and found of these that Gal-α1,6-Gal was processed to some extent but Gal-α1,4-Glc was cleaved most efficiently. To assess the catalytic proficiency of MYORG on this disaccharide, we performed more detailed kinetic analysis and were able to observe Michaelis–Menten kinetics (**Fig 2E**) that yielded values for *K*_M_ (980 ± 7 μM), *k*_cat_ (0.047 ± 0.003 min^−1^), and *k*_cat_/*K*_M_ (49 ± 7 M^−1^ min^−1^). Despite the glucose leaving group being a far worse leaving group (p*K*_a_ approximately 15) [25] as compared to 4MU (p*K*_a_ approximately 7.8), this second-order rate constant measured for Gal-α1,4-Glc is similar to that measured for 4MU-Gal. Given these collective observations, we were intrigued by the unusual substrate specificity of MYORG and set out to solve the structure of this enzyme.

### The X-ray structure of MYORG shows it is a membrane-bound dimer

Given the activity of MYORG towards α-galactosides, we set out to obtain structural insights into active site architecture to understand the molecular basis for the substrate selectivity of MYORG. In particular, we wanted to observe how differences between the active sites of MYORG and α-Glu II lead to differing substrate preferences, while also attempting to validate our kinetics data by demonstrating how selection of Gal-α1,4-Glc is achieved. We obtained crystals of unliganded MYORG and processed them in the *P*1 space group to 2.43 Å (**S2 Table**). The crystal structure was solved by molecular replacement using the *E*. *coli* GH31 enzyme YicI (PDB: 2F2H) as a search model [26]. Four copies of MYORG could be placed within the asymmetric unit (chains A to D, **S3 Fig**). For chains A to C, the polypeptide backbone could be confidently traced into the electron density map, with only the first 11 amino acids (80–91) and 2 short loop regions (residues 165–171 and 270–276) proving too disordered to model. The N-terminal β-sheet domain of chain D proved more challenging to build and residues 80–99 and 120–143 were omitted from the model due to disorder. Chain C represents the most complete model of MYORG and will be used hereafter to describe the structure of MYORG_GH31_.

**Fig 3:**
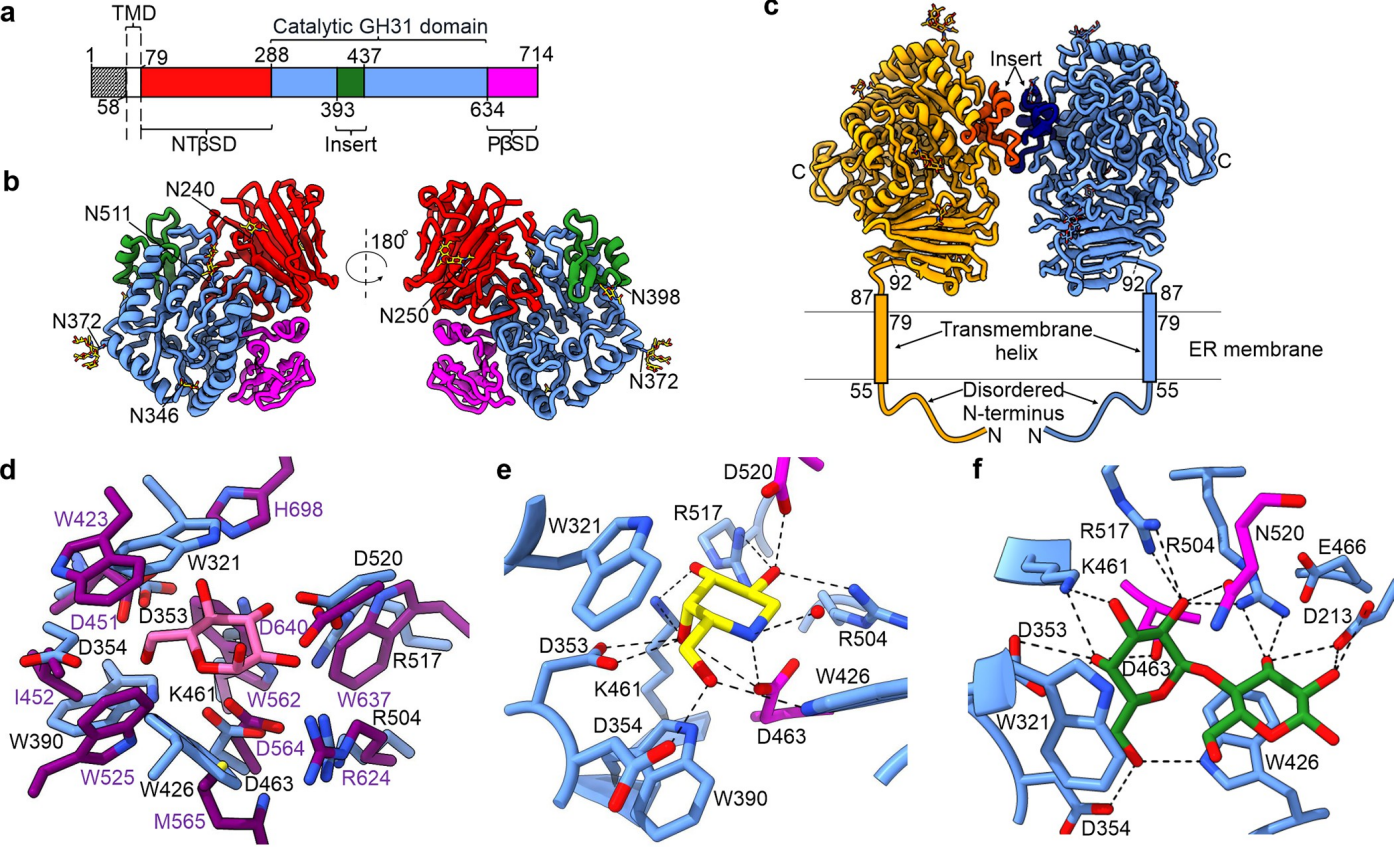
MYORG is a membrane bound dimer that selectively binds an unusual Gal-α1,4-Glc epitope. (**a**) Domain boundaries of MYORG with numbering representing the last residue of the domain. (**b**) Cartoon ribbon representation of MYORG with *N*-glycans depicted as sticks with the glycosylated Asn residues labelled. (**c**) The MYORG dimer arrangement showing the insert region and the expected orientation of MYORG with respect to the ER membrane based on analyses using PSIPRED [27], DISOPRED [28], and MEMSAT [29]. (**d**) Comparison of the active site of MYORG (blue, residue labelling in black) with that of *Mm*α-Glu-II (purple; PDB: 5H9O). D-glucose is bound by *Mm*α-Glu-II and is depicted in pink. (**e**) Residues involved in the positioning and binding of DGJ. (**f**) Residues involved in binding Gal-α1,4-Glc. Dashed lines in (**e**) and (**f**) represent hydrogen bonding. Magenta sticks are used to emphasise the catalytic acid (D520, mutated to N520 in (**f**)) and nucleophile (D463) residues. DGJ, deoxygalactonojirimycin; ER, endoplasmic reticulum; MYORG, myogenesis-regulating glycosidase; NTβSD, N-terminal β-sheet domain; PβSD, proximal β-sheet domain; TMD, transmembrane domain.

Analysis of the crystal structure reveals MYORG is comprised of an antiparallel β-sandwich N-terminal domain (residues 92–287), a (β/α)_8_-barrel catalytic domain (residues 288–633) with an insertion between α3 and α4 (residues 393–436), and a proximal β-sheet domain (residues 634–714) (**Fig 3A and 3B**). Five cysteine residues are present in the structure and form 2 disulphide bonds within the N-terminal domain (C125 with C134 and C158 with C284). DALI analysis on the isolated MYORG GH31 domain reveals greatest similarity with the *Cellvibrio japonicus* α-transglucosylase Agd31B (PDB: 5NPC, Z-score 35.1, 24% sequence identity). Unlike other eukaryotic GH31 family structures, MYORG has an elongated α8 due to formation of a π-helix turn, whereas usually a single residue linker connects α8 to the following α-helix. MYORG lacks the common distal C-terminal domain (residues 828–966 in α-Glu II), which is common in GH31 enzymes and forms a large part of the dimerization interface in the α-Glu II heterodimer.

MYORG_GH31_ is predicted to have 6 *N*-glycosylation sites: N240, N250, N346, N372, N398, and N511. Glycosylation is observed on every polypeptide chain in the asymmetric unit, and it is possible to model *N*-glycans on all of the predicted *N*-glycosylation sites of chains B and C (**Fig 3A and 3B**). The observed glycans vary in both their length and glycosylation patterns. Fucose is present linked α1,3 and α1,6 to the reducing end GlcNAc of the N372 glycan, where the former α1,3 linkage is an artefact of insect cell expression. The glycans likely extend further than can be reliably modelled, since positive difference electron density can be observed where additional residues of longer glycan chains would be found. Additional crystal contacts are likely afforded by some of these glycans, for instance, difference density emerging from glycosylated N372 within chains B and C appears to bury itself in the symmetry-related chains B and C, respectively (**S3 Fig**). Notably, numerous additional unassigned peaks in the difference map exist on the surface of the protein structures that are separated from one another by approximately 5.5 Å. These peaks are incompatible with bound water or molecules present in the crystallization conditions and may therefore represent other transiently bound glycans extending from other chains.

The crystal structure shows that MYORG forms a 2-fold dimeric assembly, agreeing with our SEC-MALLS analysis, with the protein–protein interaction exclusively localised to the insert region (**Fig 3C**). This arrangement is best represented by chains B and C; however, chains A and D also form this homodimer assembly. The interface covers 639 Å^2^ and is largely supported through a hydrophobic interaction network composed of F402, V406, L419, L422, and I429, in addition to hydrogen bonding between Y397 to E407. An *N*-glycosylation site lies near the interface (N398) and although modelled glycans do not contribute to this interface, transient interactions made through these glycans may further strengthen the interface. While the MYORG_GH31_ construct lacks the TMD and the N-terminal disordered domain, the positioning of the N-termini in relation to the dimer interface suggests the multimeric state is unlikely to be disrupted by incorporation into a membrane. Indeed, the dimer interface can be used to indicate how MYORG sits in the membrane, PSIPRED, DISOPRED, and MEMSAT analysis coupled with the crystallography data suggests only residues 88–91 are disordered, thus the catalytic domain must be angled such that its longest axis is perpendicular to the membrane (**Fig 3C**) [27–29].

Using the superimposition of MYORG onto α-Glu II (**Fig 3D**; RMSD of 2.35 Å over 495 amino acids; PDB: 5H9O), we were able to validate residues we had tentatively assigned as the nucleophile (D463) and general acid (D520) based on sequence alignments and consensus motifs (**S4 Fig**). Comparing the active site of MYORG against *Mm*α-Glu II, it is evident why MYORG is unable to accommodate glucose in the −1 subsite (**Fig 3D**). Specifically, *Mm*α-Glu II provides a space between W423 and H698 in which the 4-OH of a glucose residue can fit and hydrogen bond with H698. Whereas within the active site of MYORG, the comparable residue, W321, is positioned differently and occupies this space in a manner that would make binding of a glucose residue result in a steric clash between this residue and the 4-OH of glucose. Conversely, *Mm*α-Glu II is unable to accommodate galactose since its 4-OH would clash with W562. The equivalent residue in MYORG is K461 which would not clash and instead would likely provide a stabilising hydrogen bond.

### MYORG selectively binds α-galactosides and its substrate Gal-α1,4-Glc

To visualise how MYORG binds to DGJ, we soaked the inhibitor into crystals of wild-type MYORG_GH31_. Data was collected and processed to 2.43 Å (**S2 Table**). Strong positive *F*_*o*_*-F*_*c*_ difference density was observed in the active sites of all chains in the asymmetric unit, and DGJ was unambiguously modelled within this density in a ^4^*C*_1_ conformation (**Figs 3E and S3**). This conformation is that expected for Michaelis complexes of substrates bound to GH31 family enzymes [26] as well as, based on the structure of the *Chaetomium thermophilum* α-Glu II, for deoxynojirimycin itself [30]. MYORG makes several contacts with DGJ (**Fig 3E**). The general catalytic acid/base residue (D520) of MYORG contacts the 2-OH of DGJ, as does R504 and R517. Further interactions include hydrogen bonds between K461 and the 3-OH of DGJ, D353 with the 4-OH, and D354 and W426 with the 6-OH. The catalytic nucleophile (D463) forms a close, likely ionic interaction, with the endocyclic nitrogen of DGJ (2.59 Å). As inferred from the superimposition of MYORG onto *Mm*α-Glu II, W321 conveys specificity for galactose through its position, which would clash with the 4-OH of a glucose, mannose, or xylose unit bound in the same position (**Fig 3E**). Furthermore, the structure of the MYORG_GH31_ active site explains why we find 4MU-GalNAc, despite being galactose-configured, is not a substrate. The N-acetyl group would need to occupy the space in which the general catalytic acid residue and residues R504 and R507 are found. Indeed, the structure of the GH31 family enzyme, Nag31A from *Enterococcus faecalis* reveals a hydrophobic pocket is required in the area occupied by MYORG R504 and R507 to provide space for the GalNAc methyl group [31]. These data provide clear structural support for the strict substrate preference of MYORG for α-galactosides.

To observe how MYORG_GH31_ derives its selectivity for Gal-α1,4-Glc, we set out to capture a Michaelis complex with this disaccharide. We used a catalytically impaired variant of MYORG in which the general acid catalytic residue was conservatively mutated (D520N) to allow the intact substrate to bind stably within the active site. After obtaining crystals of this mutant enzyme, we performed ligand soaking experiments. These experiments ultimately yielded structures with unambiguous *F*_*o*_*-F*_*c*_ electron density for the substrate (**S3 Fig**). Galactose in the −1 subsite is bound in the same position and ^4^*C*_1_ conformation as seen for DGJ, with hydrogen bonding partners being identical other than the endocyclic nitrogen and D520 being swapped for oxygen and N520 (**Fig 3F**). Since the wild-type protein binds to DGJ, and the D520N mutant binds to Gal-α1,4-Glc in a near identical manner, we can be confident this is the interaction network the wild-type protein uses to bind Gal-α1,4-Glc during catalysis. The glucose residue bound in the +1 site is held in position through hydrogen bonding interactions to D213 and R504, while stacking interactions between the pyranose ring of the glucose residue and W426 likely serve to increase the overall affinity for Gal-α1,4-Glc towards MYORG.

Currently only one other family GH31 α-galactosidase structure is available, that of *Pedobacter saltans* Pedsa_3617 (*Ps*Gal31A; PDB code 4XPO) [24]. *Ps*Gal31A uses a similar positioned tryptophan (W486) to W321 to provide specificity for α-galactosides. However, the W486 of *Ps*Gal31A is positioned on a loop between β8 and α8 of the (β/α)_8_-barrel fold, whereas MYORG W321 is located on a loop between β1 (residues 317–319) and α1 (residues 330–342). Other differences include the galactose 6-OH being coordinated by D354 in MYORG, whereas *Ps*Gal31A uses Y274. Additional support is provided to the 2-OH by R517 in MYORG, for which the equivalent residue in *Ps*Gal31A, Y432, is by contrast not positioned to form bonds with the substrate. In the +1 subsite, R504 of MYORG is conserved with R418 of *Ps*Gal31A; however, W426 of MYORG is replaced by E366 in *Ps*Gal31A indicating different substrate preferences. Notably, there is no equivalent residue of D213 in *Ps*Gal31A, which is needed by MYORG to coordinate the 2-OH of glucose. Supporting these observations reflecting a different substrate preference, *Ps*Gal31A has been captured in complex with fucose at the +1 subsite.

**Fig 4:**
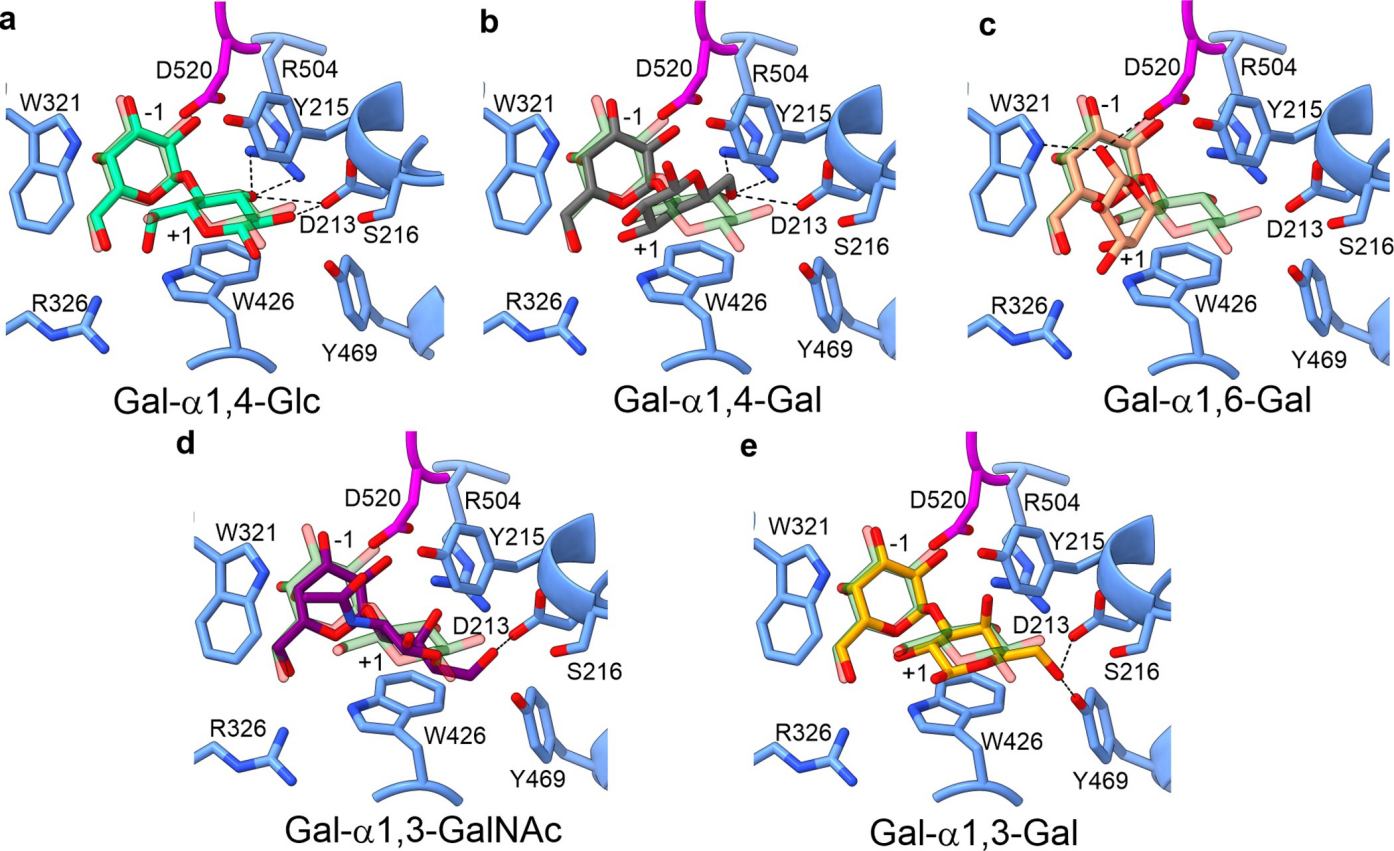
The Gal-α1,4-Glc epitope is preferentially accommodated within the active site of MYORG. (**a**) Difference in position of Gal-α1,4-Glc derived from ligand soaking experiments (dark green) from the docked model of Gal-α1,4-Glc (light green). (**b–e**) Docked natural disaccharides in the active site of MYORG. Dotted lines represent hydrogen bonding. MYORG, myogenesis-regulating glycosidase.

To determine why MYORG exhibits specificity for glucose at the +1 subsite over natural disaccharides, we performed docking experiments using AutoDock Vina [32]. Docked Gal-α1,4-Glc exhibits an almost identical position to Gal-α1,4-Glc in the crystal complex validating our docking procedure and search area (**Figs 4A and** S5). This same search area and settings were used to dock in other disaccharides. The most favourable position predicted for natural disaccharides placed the galactose residue at the −1 subsite in a near identical conformation as seen for Gal-α1,4-Glc, further validating the docking methodology (**Fig 4B–4E**). These docking studies show known disaccharides can be accommodated in the active site; however, binding of these is poor compared to glucose due to resulting steric clashes imposed by D213, W321, W426, and R504, which forced these other sugars into conformations wherein only single hydrogen bonds are formed and stacking interactions with W426 are disrupted. This reinforces our kinetic observations that currently known human substrates are unlikely to be acted upon by MYORG. In summary, the shape of the +1 subsite dictates specificity for α1,4-Glc since alternative linkages and monosaccharides bound in this site would form unproductive steric clashes.

### Structural mapping of PFBC-associated mutations

**Fig 5:**
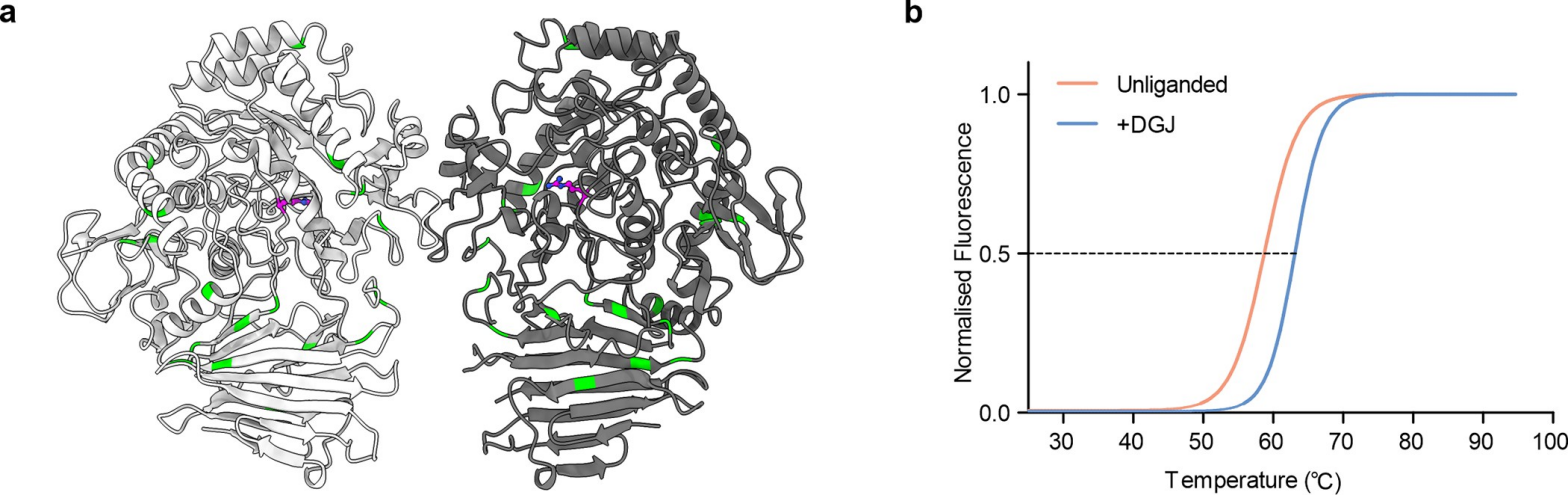
Mapping PFBC disease-associated mutations in MYORG and stabilisation of MYORG by DGJ. (**a**) Missense mutations of MYORG are depicted in green and R504 is shown as sticks in magenta. (**b**) Boltzmann fit of thermal shift data depicting difference between MYORG alone and MYORG in the presence of DGJ at a 1:20 molar ratio. Results from 3 technical replicates. All raw data underlying graphs can be found in S1 Data. DGJ, deoxygalactonojirimycin; MYORG, myogenesis-regulating glycosidase; PFBC, primary familial brain calcification.

Numerous PFBC disease-associated mutations are found within *MYORG*. We mapped these mutations onto the structure of MYORG_GH31_ and, using this model, we suggested a mechanism by which each mutation may drive disease development (**S1 Table**). While many of these mutations result in protein truncations, frameshifts, deletions, and insertions, several missense mutations are known that resulted in single amino acid changes (**Fig 5A**). Interestingly, 2 missense mutants (M35V and M35K) are found within the domain extending into the cytoplasm/nucleoplasm (**S1 Table**) and are unlikely to affect the catalytic region. As these mutations cause disease progression, it is evident that this N-terminal section is essential for homeostasis; however, how mutations of this residue lead to disease is unknown. Of the set of known mutations, only R504P has a clear impact on the active site. This mutant likely leads to loss of stabilising interactions to substrates at the −1 site and thereby disrupts substrate binding (**Fig 3**). However, introduction of a proline at this site might also cause general protein misfolding. By analogy to the many disease-associated missense mutations known to occur within various lysosomal glycoside hydrolases [33,34], other missense mutations within MYORG, including those located in the ER lumen, may lead to loss of MYORG function by causing its misfolding (**S1 Table**). Such missense mutations that cause misfolding may be amenable to treatment using small molecule pharmacological chaperones. Pharmacological chaperones have shown promise for stabilising mutant glycoside hydrolases and helping them to mature within the ER and traffic to lysosomes, as seen for the treatment of Fabry disease using DGJ (Galafold) [22,35]. However, stabilisation of proteins that are ultimately retained within the ER by promoting their proper folding and escape from the quality control pathway may also be possible. In this regard, we tested whether DGJ could stabilise MYORG, we used a thermal shift assay and found a Δ*T*_m_ of 4.4°C (**Fig 5B**). These results are in keeping with observations made using pharmacological chaperones for lysosomal enzymes and indicate that suitable chaperones may be able to promote correct folding of missense mutants of MYORG. Collectively, these observations suggest the clinically approved DGJ should be explored as a treatment option for patients with MYORG missense mutations. Such a therapy may prevent misfolding of MYORG within the ER, which could block ER-associated degradation of the mutant enzyme and increase its levels to hinder disease progression.

## Discussion

The recognized link between autosomal recessive mutations in MYORG and PFBC has stimulated increasing interest in the molecular function of this GH31-containing membrane protein. The range of mutations, which include both nonsense and missense mutations (**S1 Table**), suggests that loss of function of MYORG, rather than a gain of toxicity leads to PFBC. The sequency similarity of MYORG to the GH31 ER α-Glu II, particularly the high level of conservation of residues in the enzyme active site, coupled with their shared ER localization, [6] has led to the expectation that MYORG is an α-glucosidase [11,19] and annotation of the MYORG homologue in *Drosophila* (TOBI) as an α-glucosidase [19]. Expression of a site-directed mutant, in which the conserved active site nucleophile residue is varied, in place of the wild-type enzyme has shown that intact catalytic machinery within MYORG is needed for a normal cellular phenotype within myoblasts [11]. Showing that MYORG possesses catalytic activity has, however, been unsuccessful.

Using several lines of structural and biochemical analyses, here, we report the surprising observation that MYORG is an α-galactosidase. Since MYORG_GH31_ and TOBI share high sequence ID (86% query cover, 35% sequence identity) and structural conservation of the active site, we believe TOBI is also likely to function as an α-galactosidase, as are orthologues found within other metazoans. Moreover, the conservation of MYORG among metazoans suggests an evolutionarily conserved function, which has yet to be uncovered. From a functional perspective, within *Drosophila*, *tobi* expression is regulated by both insulin-producing cells and cells comprising the corpora cardiaca though insulin-like peptides and the insect glucagon homolog, adipokinetic hormone, which suggests a link between *tobi* and insulin signalling [19]. Indeed, expression of *tobi* can be modulated through dietary proteins and sugars, with high protein food leading to an increase in *tobi* expression, whereas high sugar food represses its expression. Reducing *tobi* expression decreases the life span of *Drosophila* on high protein food, and it is interesting that overexpression of *tobi* in flies fed a high sugar diet leads to severe growth defects and decreases in body glycogen [19]. However, the link, if any to development of PFBC in humans is unclear.

Previous functional studies on MYORG demonstrate involvement in secretion of insulin growth factor II (IGF-II) with the suggestion that MYORG interacts with pro-IGF-II [11]. Pro-IGF-II is known to be extensively *O*-glycosylated [36,37]. These observations suggested to us the possibility that MYORG might be involved in processing an α-galactosidic linkage within glycoproteins. The only 2 α-galactoside-containing glycans found to this date on glycoproteins within humans are Gal-α1,3-GalNAc that comprises the Core8 structure found in O-glycans and Gal-α1,3-Gal found within blood group antigen B. In addition, the globosides Gb2 (Gal-α1,4-Gal) and Gb3 (Gal-α1,4-Gal) are also found in humans. None of these disaccharides, however, are turned over by MYORG (**Fig 2D**). Our X-ray structure data reveals features of the MYORG active site that explain why none of these α-galactosides are turned over by this enzyme. In particular, the binary substrate complex of MYORG bound to Gal-α1,4-Glc explains both its requirement for galactosides binding within the −1 subsite and strong preference for glucose binding within the +1 binding site. Strategically positioned residues within the active site exclude the possibility of other monosaccharides binding to either of these subsites within MYORG. This substrate selectivity for Gal-α1,4-Glc is striking because glycans containing this disaccharide are currently unknown within humans. Based on these collective observations, we posit that the Gal-α1,4-Glc structure is yet to be found within the ER of mammals, where we expect it might fulfil roles in protein quality control.

The structure of the GH31 domain of MYORG enabled us to confidently map PFBC-associated mutations in MYORG. Among the 46 known disease-associated MYORG mutations, 22 are missense mutations and these can be found widely distributed throughout its GH31 domain. That such mutations, some of which are quite conservative, such as G286S and I656T, give rise to PFBC is reminiscent of the lysosomal storage diseases, where many missense mutations have been found that impair protein maturation and proper trafficking to lysosomes [38,39]. Notable in this regard is that active site ligands that bind to lysosomal enzymes [40] stabilise these proteins and facilitate their proper folding and trafficking to lysosomes. Several iminosugars have been explored as candidate therapeutics, with several having entered into the clinic [22]. Significantly, DGJ (migalastat) is clinically approved (Galafold) as a chemical chaperone for the lysosomal α-galactosidase encoded by *GLA*. We found that DGJ both bound to MYORG with reasonable affinity (*K*_D_ = 1.33 μM) and stabilised this protein against thermal denaturation. These observations raise the possibility that migalastat could be repurposed for MYORG patients having missense mutations that could be stabilised by a pharmacological chaperone. Accordingly, a logical next step will be to examine the stability of MYORG variants containing PFBC-associated missense mutations and assess the potential for migalastat to stabilise and help folding of these mutant proteins.

Finally, with regard to how MYORG contributes to PFBC, the ER localization of MYORG, coupled with its now established glycosidase activity and similarity to ER α-Glu II, makes it tempting to suggest that dysfunction of MYORG as seen in PFBC, may arise because of a role in quality control where it may regulate the folding or maturation of one or more of the protein products of genes linked to PFBC including *SLC20A2*, *PDGFB*, *PDGFRB*, and *XPR1* [2–5]. Notably, all of the products of these PFBC-linked genes are glycoproteins, and glycan processing is known to be essential for the proteolytic processing of beta subunit of platelet-derived growth factor subunit B (PDGFB) [41]. Interestingly, MYORG is only 1 of 2 PFBC-associated genes that is associated with an autosomal recessive form of this disease, the other being JAM2 [6,7], which suggests its enzymatic activity is essential to avoid downstream development of PFBC. Given these observations, a logical path forward will be to assess the effects of loss of MYORG function on the production and activity of these PFBC-associated proteins.

## Materials and methods

### Construction of wild-type and mutant MYORG expressing baculovirus

MYORG cDNA (Genscript) encoding residues 80–714 (UniProt: Q6NSJ0-1, starting V^80^SLRK) was amplified and inserted into a modified pOMNIBac plasmid (encoding N-terminal TEV protease cleavable 6xHis-tag and honey-bee melittin signal sequence) [42] by sequence and ligation-independent cloning [43,44]. Mutants of MYORG were produced using the Q5 site-directed mutagenesis kit as per manufacturer’s instructions. Recombinant bacmid was produced using a Tn7 transposition protocol in DH10EMBacY cells (Geneva Biotech) [45,46]. Bacmid was subsequently purified using a PureLink HiPure Plasmid Miniprep kit (Invitogen) by manufacturer’s instructions. To produce the V1 baculovirus stock, 6 × 2 ml volumes of 0.45 × 10^6^ cells/ml SF9 cells were each incubated statically at 28°C with 180 μl of transfection mastermix (1,050 μl Insect-XPRESS media (Lonza), 38 μl of bacmid at approximately 60 ng/μl, and 31.5 μl FuGENE HD transfection reagent (Promega)) until cells were 95% fluorescent (approximately 2 days). Cells and debris were removed by centrifugation at 200 *g* for 5 min. Fetal bovine serum was added to the clarified solution to a final concentration of 2%. To produce the V2 stock, 1 ml of V1 stock was added to 50 ml of SF9 cells at 1 × 10^6^ cells/ml, and cells were incubated at 28°C with shaking until 95% fluorescent. Cells and debris were removed by a 5-min centrifugation at 200 *g*. Fetal bovine serum was added to a final concentration of 2% to clarified conditioned media and this solution was used hereafter as the V2 stock.

### Protein expression and purification of wild-type and mutant MYORG

High Five cells (*Trichoplusia ni*) were grown to confluence of 2 × 10^6^ cell/ml (total 3.6 litres) in Gibco Express Five SFM media supplemented with 18 mM L-Glutamine before transfection with 1 ml of V2 per 600 ml of culture. At >50% cell viability and >95% fluorescence, cells and debris were removed by a 2-step centrifugation at 4°C, the first at 55 *g* for 20 min and a second at 5,500 *g* for 20 min. Conditioned media was supplemented with AEBSF (to 0.1 mM final) and imidazole (to 40 mM final). For crystallography, conditioned media was loaded onto a 5 ml HisTrap Excel column (GE Healthcare) equilibrated in buffer A (20 mM HEPES (pH 7.5), 200 mM NaCl, 40 mM imidazole, and 1 mM DTT) and eluted by a stepwise gradient of buffer B (as buffer A except 400 mM imidazole) in buffer A. Fractions containing protein were pooled and diluted 1 in 10 with 20 mM HEPES (pH 7.5), 200 mM NaCl, and 1 mM DTT before being treated 1:50 with TEV protease overnight at 4°C. Protein sample was passed over a 5 ml HisTrap Excel column equilibrated in buffer A and the flow through was concentrated using a Vivaspin centrifugal concentrator (Sartorius) and size excluded on a 16/600 Superdex 200 column (GE Healthcare) in 20 mM HEPES (pH 7.5), 200 mM NaCl, and 1 mM DTT. Protein was concentrated as before and snap frozen in liquid nitrogen.

For kinetic analysis, protein purification was modified slightly to improve purity, buffer A was swapped with buffer C (20 mM MES (pH 6.4), 200 mM NaCl, 40 mM imidazole, and 1 mM DTT) and buffer B exchanged for buffer D (as buffer C except 400 mM imidazole) for the His-tag purification. Fractions containing MYORG were pooled and dialysed overnight at 4°C with 1:50 TEV protease against 20 mM MES (pH 6.4), 50 mM NaCl, and 1 mM DTT. Dialysed samples were passed over a 1 ml HiTrap SP HP cation ion exchange column (GE Healthcare) and eluted from the column by a stepwise gradient of 20 mM MES (pH 6.4) up to 20 mM MES (pH 6.4) and 1 M NaCl. Fractions containing protein were pooled, concentrated as previously, and snap frozen in liquid nitrogen. Purity of samples was accessed by SDS-PAGE and western blot.

### Kinetics using 4MU substrates

Measurements were carried out in a 96-well black bottomed plate using a CLARIOstar plus plate reader. Assays was carried out at 25°C in a total reaction volume of 100 μl composed of reaction buffer (20 mM HEPES (pH 7.5), 200 mM NaCl, 1 mM DTT, 0.1% BSA) and 100 μM of substrate. Reaction was initiated with a final concentration of 100 nM recombinant MYORG protein in reaction buffer or reaction buffer only for the controls. Reaction was stopped at time points by adding 5 μl of reaction mix to 100 μl of stop buffer (reaction buffer adjusted to pH 10.4). Endpoint 4-methylumbelliferyl release was detected by measuring absorbance (360 nm excitation and 450 nm emission). Each reaction was run in doublet and measurements averaged. Three technical repeats were carried out for each reaction under investigation. For the pH profile, enzyme at 50 nM was assayed in 50 mM phosphate-citrate buffer (pH 5.0 to pH 8.0), 200 mM NaCl, 1 mM DTT, 0.1% BSA at 25°C. To calculate k_cat_/K_M_, enzyme at 50 nM was assayed in 20 mM MES (pH 6.5), 200 mM NaCl, 1 mM DTT, 0.1% BSA, and 2% DMSO using a substrate range of 2 mM to 31.25 μM (2-fold serial dilution) at 37°C. All rates were linear over the time course. Activity of MYORG ± DGJ was assayed in 20 mM MES (pH 6.5), 200 mM NaCl, 1 mM DTT, and 0.1% BSA at 25°C ± 10 μM DGJ. For pH, Michaelis–Menten kinetics, and MYORG ± DGJ assays, time points were taken by mixing 5 μl of reaction with 45 μl of 1 M Glycine (pH 10).

### Substrate reduction assays

The substrate scope of MYORG was assessed using the following oligosaccharides, all of which were used as received: 4-*O*-α-D-galactopyranosyl-D-glucopyranose (i.e., Gal-α1,4-Glc), Gal-α1,3-Gal, Gal-α1,4-Gal, and Gal-α1,6-Gal, were purchased from Synthose (Concord, Ontario, Canada); blood group B trisaccharide (Gal-α1,3-Gal(Fuc-α1,2)), core-8 *O*-glycan (Gal-α1,3-GalNAc), and 2′-fucosyllactose (2′FL) were obtained from Biosynth International (San Diego, California, United States of America). Stocks of all substrates were prepared in 18 MΩ·cm water and stored at −20°C until use. A total of 25 nmol of each di- or trisaccharide was mixed with 2.87 μM MYORG in 50 mM HEPES (pH 7.4), containing 50 mM NaCl and 0.025% BSA; 20 nmol 2′FL, as an internal standard, was included in all reactions. After incubating at 37°C overnight, all reactions were immediately loaded onto 250 mg Supelco ENVICarb solid phase extraction (SPE) cartridges (Sigma) that had been preconditioned by washing with 3 ml 80% methanol followed by 3 × 3 ml water. Salts and monosaccharides were washed off the SPE cartridge with water (3 ml) before any remaining oligosaccharides were eluted with 50% acetonitrile (2 × 2.2 ml). Eluted material was pooled, partially concentrated using a SpeedVac (Thermo), and lyophilized in 200-μL tubes. Samples were fluorescently labelled using 8-aminopyrene-1,3,6-trisulfonate (APTS) and analyzed by capillary electrophoresis with laser-induced fluorescence detection (CE-LIF) exactly as previously described [47]. Several representative electropherograms are depicted in **S6 Fig**. CE-LIF peak areas for all substrates were corrected against the 2′FL internal standard, and all peak ratios were subsequently normalised such that those to which no MYORG had been added were adjusted to 100%.

### Paired-enzyme assay

The kinetic parameters of MYORG against the disaccharide substrate 4-*O*-α-D-galactopyranosyl-D-glucopyranose (Gal-α1,4-Glc) were established using a glucose oxidase (GOX) and horseradish peroxidase (HRP; both from Sigma) paired-enzyme assay. The reactions were performed in a 0.1 M sodium phosphate buffer (pH 7.0). Each reaction mixture consisted of 2.25 mM 2,2′-azino-bis(3-ethylbenzothiazoline-6-sulfonic acid) diammonium salt (ABTS; Sigma), 5.40 U/ml HRP, 15.30 U/ml of GOX, and 80 nM MYORG. The substrate concentration was varied between 0 and 1,200 μM. Reactions were initiated by adding the MYORG, mixed for 2 min, and the absorbance at 575 nm was recorded at 30 s intervals over 1 h using a BioTek Epoch Microplate Spectrometer (Fisher Scientific) incubated at 25°C. Analysis was done by plotting the absorbance as a function of time in seconds. The slope for the plot (i.e., change in absorbance with time) was calculated within the range of 15 min (900 s) to 40 min (2,400 s) for each substrate concentration. The change in rate was then plotted against the substrate concentration to determine the estimated *K*_*M*_ and *V*_*max*_ of MYORG.

### Structure determination

MYORG at 10.5 mg/ml was screened against the index HT screen (Hampton Research) and diffraction quality crystals were obtained in 100 mM HEPES (pH 7.0), 10% PEG MME5000, and 5% tasimate (pH 7.0). Highest quality crystals were obtained by increasing the drop size to 3 μl (1:1 ratio protein to reservoir) and using a cat whisker to streak seed a crystal seed stock made from the same condition through it. Apo-crystals were cryoprotected in mother liqueur supplemented with 20% ethylene glycol and flash cooled in liquid nitrogen. For DGJ complexed crystals, crystals were soaked for 4 h in the same cryoprotectant supplemented with 10 mM DGJ. For the Gal-α1,4-Glc complex, D520N mutant MYORG crystals were soaked with 10 mM Gal-α1,4-Glc (Biosynth International). Diffraction data were collected at Diamond Light Source in Oxford, United Kingdom. Data reduction and processing was completed through *DIALS* and *AIMLESS* [48,49]. A structure solution was obtained through *Phaser* [50] using PDB code 2F2H as a search model after improvement using *CHAINSAW* [51]. *Phenix* AutoBuild was used to correct the sequence register in the catalytic domain by rebuilding [52]. Modelling the N-terminal domain and final cycles of refinement were completed through iterative cycles of interactive building in coot and refinement in *REFMAC5* [53,54]. Geometric restraints for DGJ were generated through *eLBOW* [55]. Restraints and validation of glycans were performed though Privateer [56]. Figures were produced in ChimeraX [57]. AutoDock Vina was used to dock substrates into the active site [32]. As the highest resolution structure and with residues primed for substrate binding, chain B of the MYORG complex with Gal-α1,4-Glc was used for docking. Before docking, N520 was reverted to D520.

### SEC-MALLS

SEC-MALLS analysis was conducted on a system comprising a Wyatt HELEOS-II multi-angle light scattering detector and a Wyatt rEX refractive index detector linked to a Shimadzu HPLC system (SPD-20A UV detector, LC20-AD isocratic pump system, DGU-20A3 degasser and SIL-20A autosampler). Experiments were conducted at ambient temperature. For protein separation, a Superdex S200 10/300 GL column pre-equilibrated in running buffer (20 mM HEPES (pH 7.5), 200 mM NaCl) was used. Sample injection was 100 μl of 3 mg/ml MYORG. Flow rate was set at 0.5 ml/min. Shimadzu LabSolutions software was used to control the HPLC and Astra 7 software for the HELEOS-II and rEX detectors. The Astra data collection was 1 min shorter than the LC solutions run to maintain synchronisation. Data were analysed using the Astra 7 software and figures created using GraphPad Prism. Molecular weights were estimated using the Zimm fit method with degree 1. A value of 0.182 was used for protein refractive index increment (dn/dc).

### Isothermal titration calorimetry

ITC measurements were taken on a MicroCal Auto-ITC_200_ calorimeter. Protein was buffer exchanged into DGJ buffer (20 mM HEPES (pH 7.0), 200 mM NaCl) using Zeba spin desalting columns, 7K MWCO as per manufacturer’s instructions. DGJ (100 μM) was added by syringe into a cell containing MYORG (10 μM) over 20, 2 μl injections (0.5 μl for first injection) at 25°C. Injections were spaced by 120 s (180 s for first injection). Binding affinity was calculated by one site fitting in the MicroCal PEAQ-ITC analysis software. Experiment was run in duplicate.

### Thermal shift analysis

A 50 μl reaction containing 20 mM MES (pH 6.5), 200 mM NaCl, 5 × SYPRO orange protein dye and 2.5 μM MYORG ± 50 μM DGJ was incrementally raised from 24°C to 94.6°C using a Stratagene Mx3005P qPCR system. Fluorescence was detected by excitation at 517 nm and emission at 585 nm. Three technical repeats were performed for each condition. Repeats were normalised to their maximum fluorescence value. To obtain a *T*_m_ value, data points were fitted with a Boltzmann model where points right of the highest value and left of the lowest value were discarded before fitting. GraphPad Prism 5 was used for fitting.

### Sequence alignments

Protein sequences were aligned through Clustal Omega [58] and figures were constructed with ESPript3 [59].

## Supporting information

S1 Fig SDS-PAGE gel displaying both fully glycosylated MYORG and MYORG after EndoH treatment. A clear reduction in size is seen upon digestion indicating removal of glycans.
(PDF)

S2 Fig Analysis of MYORG kinetics against 4MU-α-D-galactopyranoside. (**a**) Michaelis–Menten plot, k_cat_/K_M_ was estimated from linear regression analysis. Three technical replicates ± standard error. (**b**) pH activity profile of MYORG assayed in varying pH phosphate-citrate buffer. Three technical replicates ± standard error. (**c**) Activity of MYORG in the presence and absence of 10 μM DGJ. Three technical replicates ± standard deviation. All raw data underlying graphs can be found in S1 Data.
(PDF)

S3 Fig ASU contents and electron density figures. (**a**) The asymmetric unit of MYORG. Four copies of MYORG depicted as cartoon ribbons comprise the ASU. Green and yellow chains form a dimer, as do the blue and orange chains. (**b**) Transient crystal contacts likely provided by extension of the N-glycosylated N372 glycan. *F*_*o*_*-F*_*c*_ (green mesh, 3 σ contour) and 2*F*_*o*_*-F*_*c*_ (blue mesh, 1 σ contour) electron density generated from the final model indicate the last modelled GlcNAc unit is extended further; however, the electron density is too diffuse to accurately model further sugar units. SR, symmetry-related chain. (**c**) Omit *F*_*o*_*-F*_*c*_ electron density map for DGJ contoured to 3 σ. DGJ is superimposed onto the density to indicate placement. (**d**) Omit *F*_*o*_*-F*_*c*_ electron density for Gal-α1,4-Glc contoured to 3 σ. Gal-α1,4-Glc is superimposed onto the density to indicate placement.
(PDF)

S4 Fig Sequence alignment of MYORG against other CAZy GH31 family enzymes. Sequence numbered relative to MYORG. Orange double asterisk indicates nucleophile residue. Green double asterisk indicates acid/base residue. UniProt identifiers: Q6NSJ0 (*Hs*MYORG), A0A0F7R6D6 (*Ps*Gal31A), P32138 (*Ec*YihQ), P31434 (*Ec*YicI), A5FBI1 (*Fj*Dex31A), and Q8BHN3 (*Mm*α-Glu II).
(PDF)

S5 Fig The region of MYORG used for docking simulations. Chain B of MYORG from the MYORG-Gal-α1,4-Glc complex was used for docking. Docking region enclosed in green square. Acid/base and nucleophile residue coloured in magenta.
(PDF)

S6 Fig Representative CE-LIF electropherograms for candidate MYORG substrates. MYORG-active substrate (i) Gal-α1,4-Glc, MYORG-resistant substrate (ii) blood group B trisaccharide, and (iii) 2′-fucosyllactose, which was included as an internal standard in all reactions. Peaks denoted with an asterisks (*) are due to excess fluorogenic reagents. Note that monosaccharides are lost during the desalting process prior to fluorescent labelling.
(PDF)

S1 Table Mutations identified in MYORG that cause PFBC and the associated structural consequences. (PDF)

S2 Table Data collection and refinement statistics for MYORG. Values in parenthesis are for highest-resolution shell. Each dataset was derived from a single crystal.
(PDF)

S3 Table Isothermal titration calorimetry results for MYORG. Values are the mean of 2 technical repeats ± standard deviations.
(PDF)

S1 Raw Images Original unmodified gel picture taken for S1 Fig. (PDF)

S1 Data Raw data underlying graphs. (XLSX)

## Abbreviations

CE-LIF: capillary electrophoresis with laser-induced fluorescence detection
DGJ: deoxygalactonojirimycin
ER: endoplasmic reticulum
GH: glycoside hydrolase
GOX: glucose oxidase
HRP: horseradish peroxidase
MYORG: myogenesis-regulating glycosidase
PDGFB: platelet-derived growth factor subunit B
PFBC: primary familial brain calcification
SPE: solid phase extraction
TMD: transmembrane domain
