# Supplementary material for: The primary familial brain calcification-associated protein MYORG is an α-galactosidase with restricted substrate specificity
Source: PLoS Biol. 2022 Sep 21;20(9):e3001764. doi: 10.1371/journal.pbio.3001764 (PMC9491548; doi:10.1371/journal.pbio.3001764)
Supplement: S1 Fig — A clear reduction in size is seen upon digestion indicating removal of glycans. (PDF) [file pbio.3001764.s001.pdf]

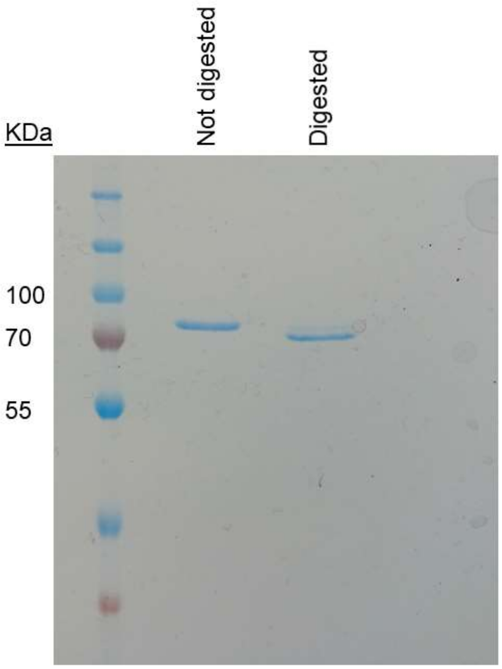

Figure S1. SDS-PAGE gel displaying both fully glycosylated MYORG and MYORG after EndoH treatment. A clear reduction in size is seen upon digestion indicating removal of glycans.
