# Supplementary material for: The primary familial brain calcification-associated protein MYORG is an α-galactosidase with restricted substrate specificity
Source: PLoS Biol. 2022 Sep 21;20(9):e3001764. doi: 10.1371/journal.pbio.3001764 (PMC9491548; doi:10.1371/journal.pbio.3001764)
Supplement: S2 Fig — (a) Michaelis–Menten plot, kcat/KM was estimated from linear regression analysis. Three technical replicates ± standard error. (b) pH activity profile of MYORG assayed in varying pH phosphate-citrate buffer. Three technical replicates ± standard error. (c) Activity of MYORG in the presence and absence of 10 μM DGJ. Three technical replicates ± standard deviation. All raw data underlying graphs can be found in S1 Data. (PDF) [file pbio.3001764.s002.pdf]

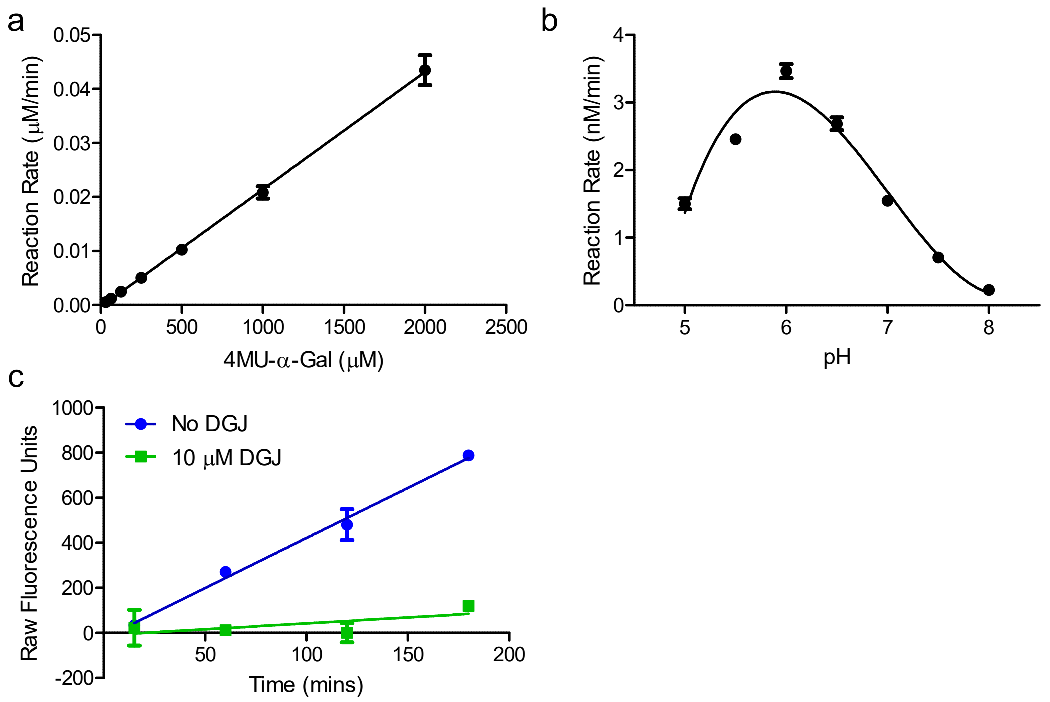

Figure S2. Analysis of MYORG kinetics against 4MU-α-D-galactopyranoside. a, Michaelis-Menten plot, k_{cat}/K_M was estimated from linear regression analysis. Three technical replicates ± standard error. **b,** pH activity profile of MYORG assayed in varying pH phosphate-citrate buffer. Three technical replicates ± standard error. **c**, Activity of MYORG in the presence and absence of 10 μM DGJ. Three technical replicates ± standard deviation. All raw data underlying graphs can be found in S1_Data.
