# Supplementary material for: The primary familial brain calcification-associated protein MYORG is an α-galactosidase with restricted substrate specificity
Source: PLoS Biol. 2022 Sep 21;20(9):e3001764. doi: 10.1371/journal.pbio.3001764 (PMC9491548; doi:10.1371/journal.pbio.3001764)
Supplement: S3 Fig — (a) The asymmetric unit of MYORG. Four copies of MYORG depicted as cartoon ribbons comprise the ASU. Green and yellow chains form a dimer, as do the blue and orange chains. (b) Transient crystal contacts likely provided by extension of the N-glycosylated N372 glycan. Fo-Fc (green mesh, 3 σ contour) and 2Fo-Fc (blue mesh, 1 σ contour) electron density generated from the final model indicate the last modelled GlcNAc unit is extended further; however, the electron density is too diffuse to accurately model further sugar units. SR, symmetry-related chain. (c) Omit Fo-Fc electron density map for DGJ contoured to 3 σ. DGJ is superimposed onto the density to indicate placement. (d) Omit Fo-Fc electron density for Gal-α1,4-Glc contoured to 3 σ. Gal-α1,4-Glc is superimposed onto the density to indicate placement. (PDF) [file pbio.3001764.s003.pdf]

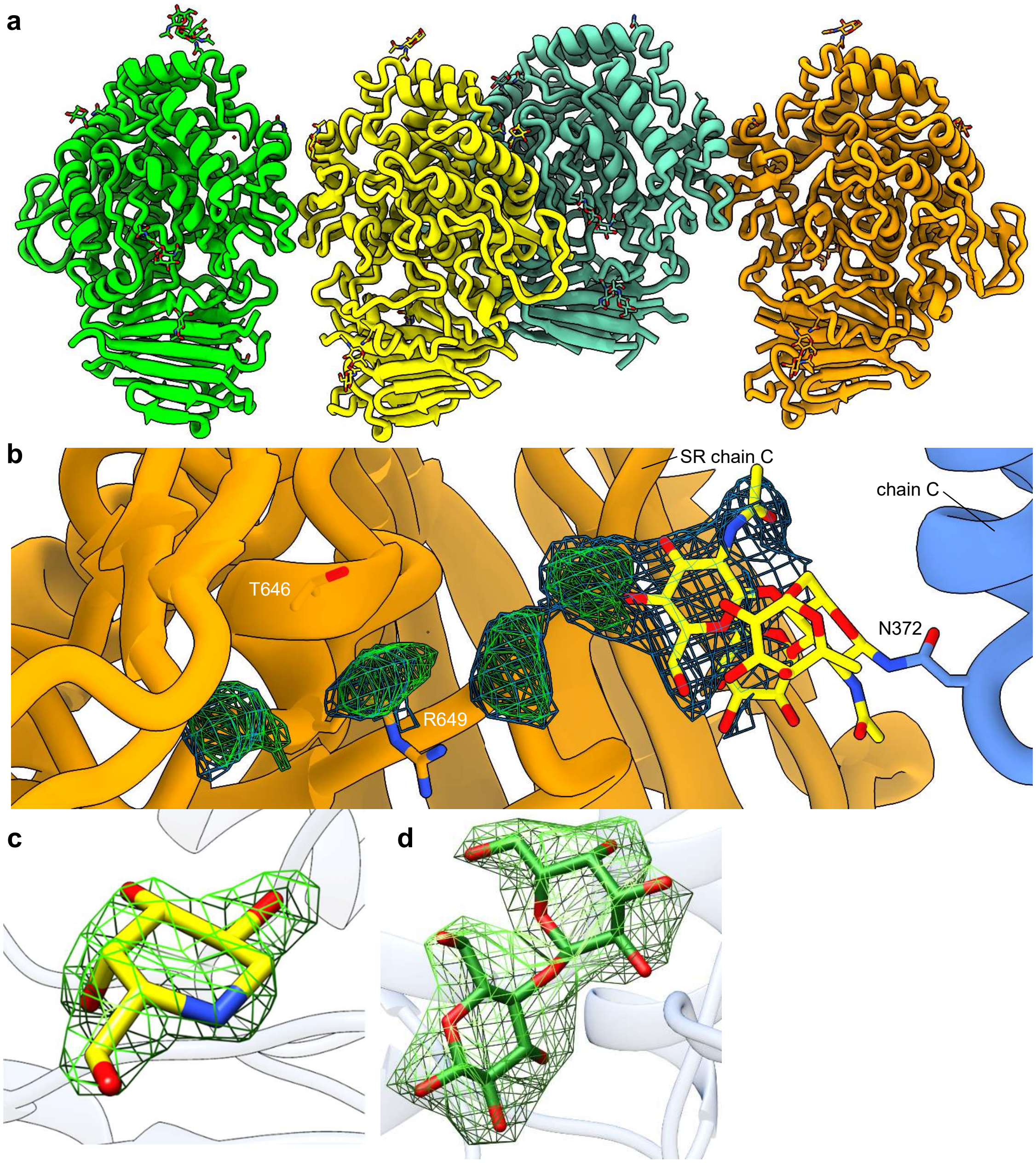

Figure S3. ASU contents and electron density figures. a, the asymmetric unit of MYORG. Four copies of MYORG depicted as cartoon ribbons comprise the ASU. Green and yellow chains form a dimer, as do the blue and orange chains. **b,** Transient crystal contacts likely provided by extension of the N-glycosylated N372 glycan. F_o-F_c (green mesh, 3 σ contour) and 2F_o-F_c (blue mesh, 1 σ contour) electron density generated from the final model indicate the last modelled GlcNAc unit is extended further, however the electron density is too diffuse to accurately model further sugar units. SR, symmetry-related chain. **c,** Omit F_o-F_c electron density map for DGJ contoured to 3 σ. DGJ is superimposed onto the density to indicate placement. **d,** Omit F_o-F_c electron density for Gal-α1,4-Glc contoured to 3 σ. Gal-α1,4-Glc is superimposed onto the density to indicate placement.
