# Supplementary material for: The primary familial brain calcification-associated protein MYORG is an α-galactosidase with restricted substrate specificity
Source: PLoS Biol. 2022 Sep 21;20(9):e3001764. doi: 10.1371/journal.pbio.3001764 (PMC9491548; doi:10.1371/journal.pbio.3001764)
Supplement: S4 Fig — Sequence numbered relative to MYORG. Orange double asterisk indicates nucleophile residue. Green double asterisk indicates acid/base residue. UniProt identifiers: Q6NSJ0 (HsMYORG), A0A0F7R6D6 (PsGal31A), P32138 (EcYihQ), P31434 (EcYicI), A5FBI1 (FjDex31A), and Q8BHN3 (Mmα-Glu II). (PDF) [file pbio.3001764.s004.pdf]

```
                                                      1       10
HsMYORG      .........................................MLQNP.QEKSQAYPRRRRP
PsGal31A     ............................................................
EcYihQ       ............................................................
EcYicI       ................................................MKISDGNWLIQP
FjDex31A     MKKSIFYCCVIFCIMVSQAYSQKKEQYLGNCTAYSVKGNKVVFSCANNSKI.......M.
Mm_aGlu-II   MA.....AIAAVAARRRRSWLSLVLAYLGVCLGITLAVDRSNFKTCDESSFCKRQRSIRP

             20        30                                  40
HsMYORG      GCYAYRQNPEAIA..........................AAAMYTFLP............
PsGal31A     ............................................................
EcYihQ       ............................................................
EcYicI       GLNLIH.......................PLQVFEVEQQDNEMVVYA....APRDVRERT
FjDex31A     ...........LQLCS........GEVVKIWAS.....ADGNFVR........NNESFAV
Mm_aGlu-II   GLSPYRALLDTLQLGPDALTVHLIHEVTKVLLVLELQGLQKNMTRIRIDELEPRRPRYRV

                                                      50        60
HsMYORG      .............DNFSPAKPK....................PSKDLKPLLGSAVLGLLL
PsGal31A     ...............................MN......KFKKLIQLFCLLFVA.LFYTS
EcYihQ       .....MDTPRPQLLDFQ.......................FHQNNDSFTLHFQQRLILTH
EcYicI       ...WQLDTPLFTLRFFSPQEGIVGV.....RIEHFQGA...LNNGPHYPLNILQDVKVTI
FjDex31A     IE........EDLGW....KGNVTVKEEPSTYEIFTEQLRIRVNKAPFQLQIFDKYQKLL
Mm_aGlu-II   PDVLVADPPTARLSV....SGR...DDNSVELTVAEGPYKIILTAQPFRLDLLEDRSLLL

              70           80        90       100
HsMYORG      VLAAVVAWCY...YSVSLRKAERLRAELLDLKAGGFSIRNQKGE................
PsGal31A     SHAQTANWTEIYPGV...WKATVGKPESYDLLKAAGAQPNKD..............ALSK
EcYihQ       SKDNPCLWIGSGIADIDMF.............RGNFSIKDK....LQEKIALTDAIVSQS
EcYicI       ENT.......ERYAEFKS...............GNL..................SARVSK
FjDex31A     FSD....YAEKGFVN..................DNG........................
Mm_aGlu-II   SVN......ARGLMAFEHQRAPRVPQESKDPAEGNGAQPEATPGDGDKPEETQEKAEKDE

             110       120                                     130
HsMYORG      .QVFRLAFRSGALDLD..............................SCSRDGALLGCSLT
PsGal31A     TEKVSFPFANGGVS....................LEVSGGKTYLRFPLQKEEQLYGFGLN
EcYihQ       PDGWLIHFSRGSDISATLNISADDQGRLLLELQNDNLNHNRIWLRLAAQPEDHIYGCGEQ
EcYicI       GEFWSLDFLRNGERITGSQVKN......NGYVQDTNNQRNYMFERLDLGVGETVYGLGER
FjDex31A     ........................................KIRTNKVLRNDEQFFGLGEK
Mm_aGlu-II   PGAWEETFKTHSDS......K........PY......GPTSVGLDFSLPGMEHVYGIPEH

             140                150       160       170       180
HsMYORG      ADG.........LPLHFFIQTVRPKDTVMCYRVRWEEAAPGRAVEHAMFLGDAAAHWYGG
PsGal31A     FQT....VHQRGKILELHVD......H...YGGK..........................
EcYihQ       FSY....FDLRGKPFPLWTS......EQGVGRNK..........................
EcYicI       FTA....LVRNGQTVETWNR......DGG...T...........................
FjDex31A     SGN....LNRRGSAYKMWNS......DQPCYGVN..........................
Mm_aGlu-II   ADSLRLKVTEGGEPYRLYNL......DVFQYELN..........................

            190       200              210       220       230        240
HsMYORG      AEMRTQHWPIRLD........GQQEPQPFVTSDVYSSDAAFGGILERYWLSSRAAAIKVN
PsGal31A     .D.SGRT................HAPTPFYVSS...N..GYG....VFINSARYIK....
EcYihQ       .Q.TYVTWQADCKENAGGDYYWTFFPQPTFVST...Q..KYY....CHVDNSCYMN....
EcYicI       .S.TEQA................YKNIPFYMTN...R..GYG....VLVNHPQCVS....
FjDex31A     .E..DPL................YKSIPFFMSS...Y..RYG....IFFDNTYKTE....
Mm_aGlu-II   .N.PMAL................YGSVPVLLAHSFHR..DLG....IFWLNAAETW....

                    250          260            270        280       290
HsMYORG      DSVPFHLGWNSTERSLRLQ...ARY.....HDTPYKPP.AGRAAAPELSYRVCVGSDVTS
PsGal31A     ....VWAGTGVRKDSENFPTPKDRNTDKTWSSRPYSDAVEILVPAEGVEVYVFGGPKPID
EcYihQ       ....FDFSAPEY.......................HELALWEDK...ATLRFECADTYIS
EcYicI       ....FEVG........................SEKVSKVQFSVESEYLEYFVIDGPTPKA
FjDex31A     ....FKFG........................SESNDYYSFEAPAGQMVYYFMFGNDYKE
Mm_aGlu-II   ....VDISSNTAGKT.LFGKMLDYL...QGSGETPQTDIRWMSESGIIDVFLMLGPSVFD

                   300       310       320       330       340       350
HsMYORG      IHKYMVRRYFNKPSRVPAPEAFRDPIWSTWALYGRAVDQDKVLRFAQQIRLHHFNSSHLE
PsGal31A     ....AVKRYNLLNGGGYLPPRWGLGFTQRVM...TRYTDKDVEKEVNDFKEKGYPLDFVG
EcYihQ       ....LLEKLTALLGRQPELPDWIYDGVTLGI..QG..GTEVCQKKLDTMRNAGVKVNGIW
EcYicI       ....VLDRYTRFTGRPALPPAWSFGLWLTTS.FTTNYDEATVNSFIDGMAERNLPLHVFH
FjDex31A     ....IIQNYIALTGKPIMPPKWALGFSQCRG...DYTREDQAREIAAEFRKRKIPCDIIY
Mm_aGlu-II   ....VFRQYASLTGTQALPPLFSLGYHQSRW...NYRDEADVLEVDQGFDDHNMPCDVIW

                           360       370       380       390       400
HsMYORG      IDD........MYTPAYGDFDFDEVKFPNASDMFRRLRDAGFRVTLWVHPFVNYNSS..R
PsGal31A     LEP....GWQS..KAYPGTFSWDKSRYPDPTSFVKKMKDQGIRLNLWINPYISPDAPF..
EcYihQ       AQDWSGIRMTSFGKRVMWNWKWNSENYPQLDSRIKQWNQEGVQFLAYINPYVASDKD..L
EcYicI       FDC....FW..MKAFQWCDFEWDPLTFPDPEGMIRRLKAKGLKICVWINPYIGQKSP..V
FjDex31A     QDI....GW..TEGLQ..DFDWRKNNYNNPKGMVKDLSDMGFKMIVSQDPVISQANQQQW
Mm_aGlu-II   LDI....EH..ADGKR..YFTWDPTRFPQPLNMLEHLASKRRKLVAIVDPHIKVDSGYRV
```

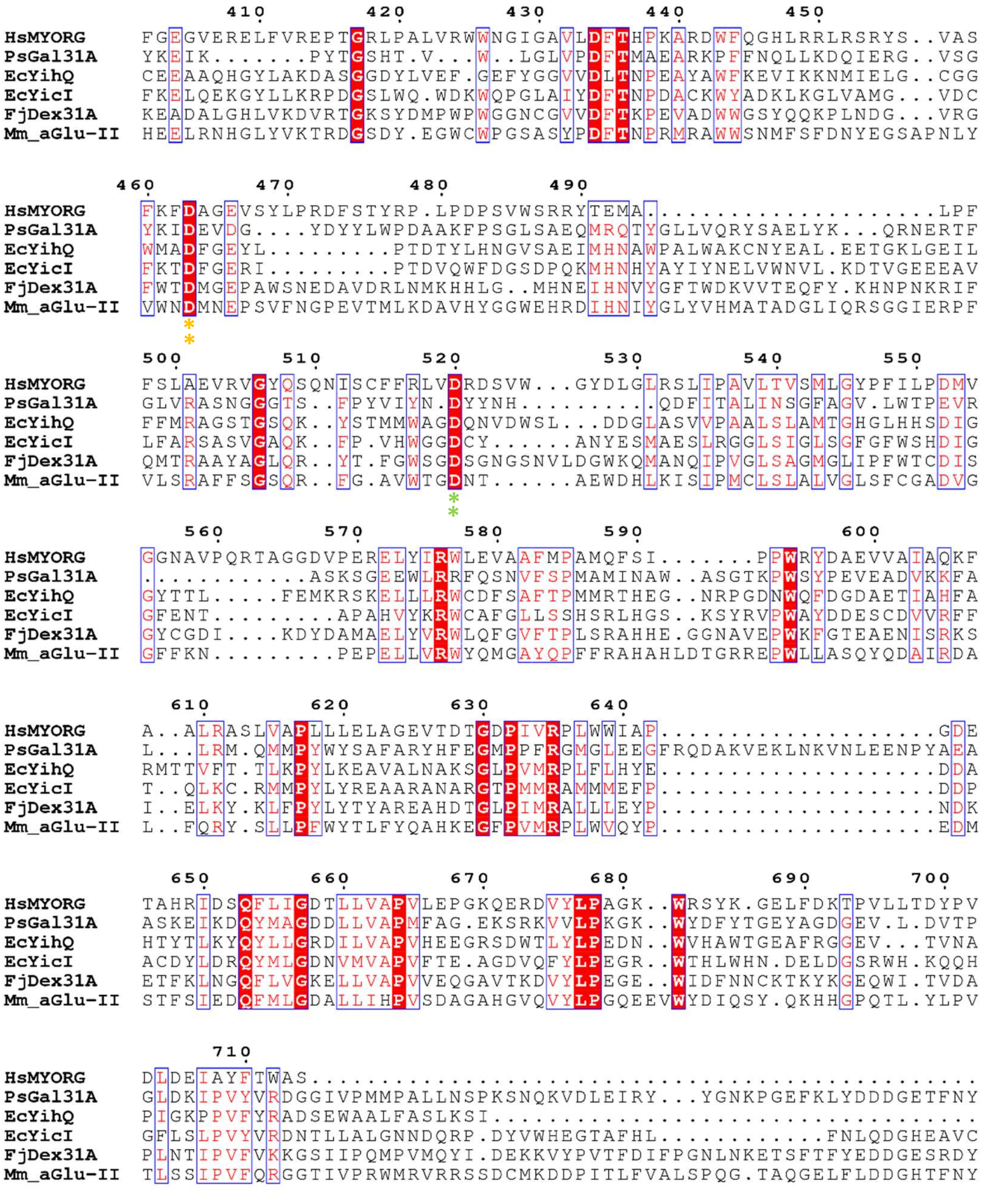

Figure S4. Sequence alignment of MYORG against other CAZy GH31 family enzymes. Sequence numbered relative to MYORG. Orange double asterisk indicates nucleophile residue. Green double asterisk indicates acid/base residue. UniProt identifiers: Q6NSJ0 (*Hs*MYORG), A0A0F7R6D6 (*Ps*Gal31A), P32138 (*Ec*YihQ), P31434 (*Ec*YicI), A5FBI1 (*Fj*Dex31A) and Q8BHN3 (*Mm*α-Glu II).
