# Supplementary material for: The primary familial brain calcification-associated protein MYORG is an α-galactosidase with restricted substrate specificity
Source: PLoS Biol. 2022 Sep 21;20(9):e3001764. doi: 10.1371/journal.pbio.3001764 (PMC9491548; doi:10.1371/journal.pbio.3001764)
Supplement: S5 Fig — Chain B of MYORG from the MYORG-Gal-α1,4-Glc complex was used for docking. Docking region enclosed in green square. Acid/base and nucleophile residue coloured in magenta. (PDF) [file pbio.3001764.s005.pdf]

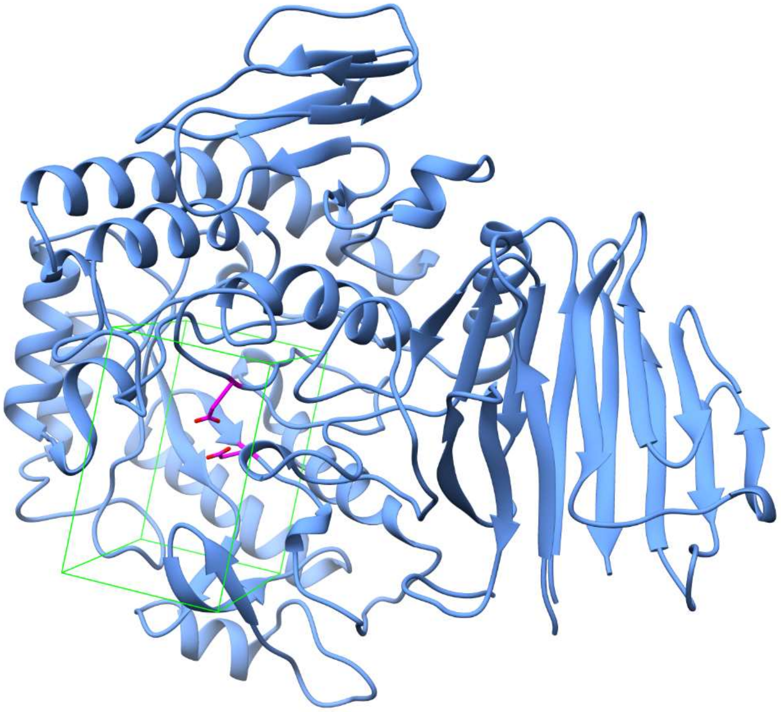

Figure S5. The region of MYORG used for docking simulations. Chain B of MYORG from the MYORG-Gal-α1,4-Glc complex was used for docking. Docking region enclosed in green square. Acid/base and nucleophile residue coloured in magenta.
