# Supplementary material for: The primary familial brain calcification-associated protein MYORG is an α-galactosidase with restricted substrate specificity
Source: PLoS Biol. 2022 Sep 21;20(9):e3001764. doi: 10.1371/journal.pbio.3001764 (PMC9491548; doi:10.1371/journal.pbio.3001764)
Supplement: S6 Fig — MYORG-active substrate (i) Gal-α1,4-Glc, MYORG-resistant substrate (ii) blood group B trisaccharide, and (iii) 2′-fucosyllactose, which was included as an internal standard in all reactions. Peaks denoted with an asterisks (*) are due to excess fluorogenic reagents. Note that monosaccharides are lost during the desalting process prior to fluorescent labelling. (PDF) [file pbio.3001764.s006.pdf]

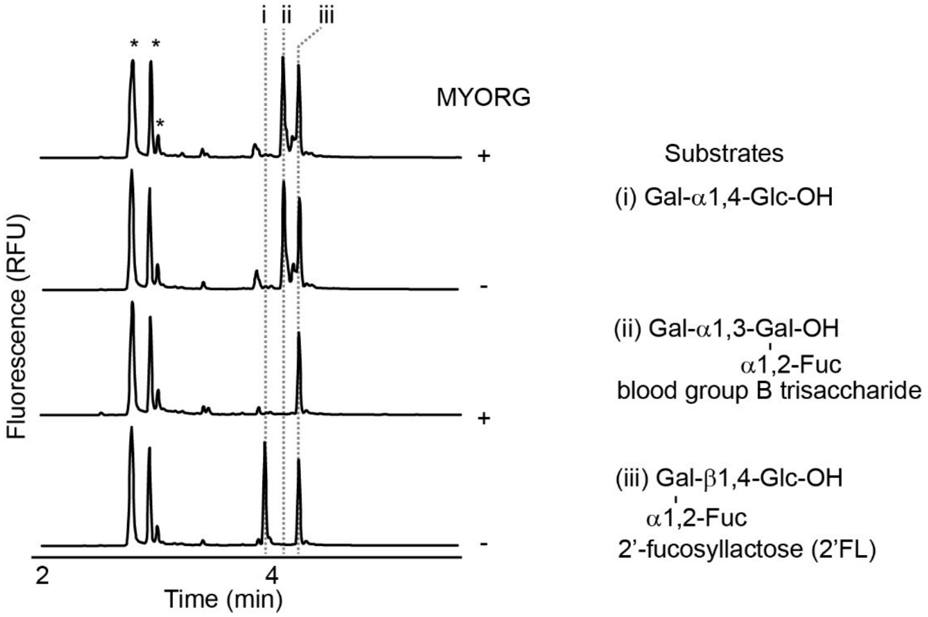

Figure S6. Representative CE-LIF electropherograms for candidate MYORG substrates. MYORG-active substrate (i) Gal-α1,4-Glc, MYORG-resistant substrate (ii) blood group B trisaccharide, and (iii) 2'-Fucosyllactose, which was included as an internal standard in all reactions. Peaks denoted with an asterisks (*) are due to excess fluorogenic reagents. Note that monosaccharides are lost during the desalting process prior to fluorescent labelling.
