# Supplementary material for: The primary familial brain calcification-associated protein MYORG is an α-galactosidase with restricted substrate specificity
Source: PLoS Biol. 2022 Sep 21;20(9):e3001764. doi: 10.1371/journal.pbio.3001764 (PMC9491548; doi:10.1371/journal.pbio.3001764)
Supplement: S1 Table — (PDF) [file pbio.3001764.s007.pdf]

Table S1. Mutations identified in MYORG that cause PFBC and the associated structural consequences.

DNA change	Amino acid change	Location	Effect	Original Reference
c.103 A>G	M35V	N-terminal region	- Unknown	(1)
c.104 T>A	M35K	N-terminal region	- Unknown	(2)
c.191 G>A	G64E	Transmembrane helix	- Unknown	(3)
c.225 G>A	W75*	Transmembrane helix	- Severe truncation of protein.	(1)
c.285-310 del insert TTC	L95 fs	N-terminal domain	- Incorrect protein sequence from L95	(4)
c.337-348 dup	L113-R116 dup	N-terminal domain	- General disruption of protein fold.	(5)
c.338 T>G	L113R	N-terminal domain	- Disruption of hydrophobic core through steric clashes, caused by introduction of a large, charged sidechain.	(3)
c.348-349 insert CTGGCCTTCCGC	116-117 insert LAFR	N-terminal domain	- General disruption of protein fold.	(1)
	125-132 del CSRDGALL and insert	N-terminal domain	- Loss of stabilising di-sulphide bond.<br>- General disruption of protein fold	(6)
c.428_442 del TGCACTTCTTCATCC	143_147 del LHFFI	N-terminal domain	- General disruption of protein fold	(7)
c.535-536 insert C	G179 fs	N-terminal domain	- Incorrect protein sequence from G179	(4)
c.607 C>T	Q203*	N-terminal domain	- Severe truncation of protein	(1)
c.679 C>G	R227G	N-terminal domain	- Loss of hydrogen bonding sidechain. R227 likely to aid in positioning of the loop carrying D213 (possible involved in binding polysaccharides at active site)	(8)
c.687 G>T	W229C	N-terminal domain	- Disruption of stabilising hydrophobic core.	(7)
c.695 C>T	S232L	N-terminal domain	- General disruption of structure through introduction of a larger sidechain causing steric clashes.	(1)
c.706_708 dup GCC	A236 dup	N-terminal domain	- Insertion of additional residue into β-sheet.	(3)
c.747G>C	W249C	N-terminal domain	- Disruption of stabilising hydrophobic core.	(3)

c.782-783 GC>TT	R261L	N-terminal domain	- Disruption of hydrogen bonding involved in the positioning of the loop carrying the likely active site residue D213.<br>-Introduction of hydrophobic facing solvent	(1)
c.782 G>T	R261L			(8)
c.794 C>T	T265M	N-terminal domain	-Introduction of a bulky hydrophobic residue causing loop disruption.	(9)
c.830 del C	P277 fs	N-terminal domain	- Incorrect protein sequence from P277	(10)
c.850 T>C	C284R	N-terminal domain	-Breaks a disulphide bond and introduces a bulky charged residue causing fold disruption.	(2)
c.854_855 dup TG	G286W fs	N-terminal domain	- Loss of catalytic domain	(11)
c.856 G>A	G286S	N-terminal domain	-possible disruption of hydrophobic network affecting N-terminal domain fold.	(12)
c.893 G>C	R298P	Catalytic domain	- Disruption of α-helix	(8)
c.940 C>T	R314*	Catalytic domain	- Severe truncation of catalytic domain	(6)
c.1060_1062delGAC	354 del D	Catalytic domain	- D354 Is found in an active site loop, mutation would disrupt bonding of D353 to substrate and may also cause larger, global structural changes to MYORG through residue loss.	(13)
c.1078 del T	Y360 fs	Catalytic domain	- Loss of catalytic domain	(3)
c.1092-1097delCTTCGA	365-366 del FD	Catalytic domain	- F365 forms part of a hydrophobic core<br>- General disruption of protein fold	(1)
c.1118 C>A	A373D	Catalytic domain	- Would cause steric clash leading to α-helix displacement.<br>- May make N372 unavailable for glycosylation, possibly affecting protein production.	(3)
c.1233delC	F411L + fs	Insert	- Loss of catalytic domain.	(13)
c.1300 G>C	D434H	Insert	- Introduction of severe steric clashes.	(3)
c.1321 C>G	R441G	Catalytic domain	- Loss of stabilising salt bridge.	(1)

c.1328 G>A	W443*	Catalytic domain	- Loss of catalytic domain	(1)
c.1333 C>T	Q445*	Catalytic domain	- Loss of catalytic domain	(14)
c.1394 dup G	E466 fs	Catalytic domain	- Loss of catalytic domain	(3)
c.1427 C>A	T476N	Catalytic domain	- Likely to cause steric clashes.	(3)
c.1431 C>A	Y477*	Catalytic domain	- Loss of catalytic domain	(7)
c.1530 del G	N511T fs	Catalytic domain	- Loss of catalytic domain	(15)
c.1511 G>C	R504P	Catalytic domain	- Conserved residue associated with binding sugar substrates.	(8)
c.1538_1540 del CCT	513 del S	Catalytic domain	-Loss of stabilising hydrogen bond.<br>- Would alter positioning of N511 possibly hindering glycosylation and protein processing.<br>- Global structural issues likely from loss of amino acid.	(3)
c.1802_1804 del TGG	601 del V	Catalytic domain	- Loss of amino acid in α-helix affecting register/fold.	(8)
c.1831 C>T	R611W	Catalytic domain	- Disruption of important hydrogen bonding network.<br>- Severe steric clashes introduced.	(3)
c.1865 T>C	L622P	Catalytic domain	- Disruption of α-helix	(3)
c.1967 T>C	I656T	Proximal β-sheet domain	- Disruption of stabilising hydrophobic core, anchoring proximal β-sheet domain to catalytic domain.	(16)
c.1979 T>A	L660Q	Proximal β-sheet domain	- Disruption of stabilising hydrophobic core, anchoring proximal β-sheet domain to catalytic domain.	(3)

References

1. Yao XP, Cheng X, Wang C, Zhao M, Guo XX, Su HZ, et al. Biallelic Mutations in MYORG Cause Autosomal Recessive Primary Familial Brain Calcification. Neuron. 2018;98(6):1116-23.e5.
2. Chen SY, Lin WC, Chang YY, Lin TK, Lan MY. Brain hypoperfusion and nigrostriatal dopaminergic dysfunction in primary familial brain calcification caused by novel MYORG variants: case report. BMC Neurol. 2020;20(1):329.
3. Grangeon L, Wallon D, Charbonnier C, Quenez O, Richard AC, Rousseau S, et al. Biallelic MYORG mutation carriers exhibit primary brain calcification with a distinct phenotype. Brain. 2019;142(6):1573-86.
4. Malaquias MJ, Martins RC, Oliveira J, Freixo JP, Magalhães M. MYORG gene disease-causing variants in a family with primary familial brain calcification presenting with stroke-like episodes. Clin Genet. 2020;98(5):517-9.
5. Du J, Zhu X, Liu J, Tan Y. Paroxysmal Kinesigenic Dyskinesia Secondary to Brain Calcification with a Homozygous MYORG Mutation. Mov Disord. 2021.
6. Taglia I, Kuipers DJS, Breedveld GJ, Mignarri A, Dotti MT, Federico A, et al. Primary familial brain calcification caused by MYORG mutations in an Italian family. Parkinsonism Relat Disord. 2019;67:24-6.
7. Chen Y, Fu F, Chen S, Cen Z, Tang H, Huang J, et al. Evaluation of MYORG mutations as a novel cause of primary familial brain calcification. Mov Disord. 2019;34(2):291-7.
8. Chen Y, Cen Z, Chen X, Wang H, Chen S, Yang D, et al. MYORG Mutation Heterozygosity Is Associated With Brain Calcification. Mov Disord. 2020;35(4):679-86.
9. Kume K, Takata T, Morino H, Matsuda Y, Ohsawa R, Tada Y, et al. The first Japanese case of primary familial brain calcification caused by an MYORG variant. J Hum Genet. 2020;65(10):917-20.
10. Gao L, Chen J, Dong H, Li X. A novel mutation in MYORG leads to primary familial brain calcification and cerebral infarction. Int J Neurosci. 2021:1-5.
11. Ferreira LD, de Oliveira JRM. New homozygous indel in MYORG linked to brain calcification, thyroidopathy and neuropathy. Brain. 2019;142(9):e51.
12. Tekin Orgun L, Besen Ş, Sangün Ö, Bisgin A, Alkan Ö, Erol İ. First pediatric case with primary familial brain calcification due to a novel variant on the MYORG gene and review of the literature. Brain Dev. 2021;43(7):789-97.
13. Arkadir D, Lossos A, Rahat D, Abu Snineh M, Schueler-Furman O, Nitschke S, et al. MYORG is associated with recessive primary familial brain calcification. Ann Clin Transl Neurol. 2019;6(1):106-13.
14. Peng Y, Wang P, Chen Z, Jiang H. A novel mutation in MYORG causes primary familial brain calcification with central neuropathic pain. Clin Genet. 2019;95(3):433-5.
15. Ramos EM, Roca A, Chumchim N, Dokuru DR, Van Berlo V, De Michele G, et al. Primary familial brain calcification caused by a novel homozygous MYORG mutation in a consanguineous Italian family. Neurogenetics. 2019;20(2):99-102.
16. Forouhideh Y, Müller K, Ruf W, Assi M, Seker T, Tunca C, et al. A biallelic mutation links MYORG to autosomal-recessive primary familial brain calcification. Brain. 2019;142(2):e4.
