# Supplementary material for: The primary familial brain calcification-associated protein MYORG is an α-galactosidase with restricted substrate specificity
Source: PLoS Biol. 2022 Sep 21;20(9):e3001764. doi: 10.1371/journal.pbio.3001764 (PMC9491548; doi:10.1371/journal.pbio.3001764)
Supplement: S2 Table — Values in parenthesis are for highest-resolution shell. Each dataset was derived from a single crystal. (PDF) [file pbio.3001764.s008.pdf]

Table S2. Data collection and refinement statistics for MYORG. Values in parenthesis are for highest-resolution shell. Each dataset was derived from a single crystal.

	Unliganded	DGJ complex	Gal-α1,4-Glc complex
Accession code	7QQF	7QQG	7QQH
**Data Collection**			
Spacegroup	*P*1	*P*1	*P*1
Cell dimensions			
a, b, c (Å)	74.1, 79.0, 180.1	73.1, 78.9, 176.2	73.3, 79.1, 178.0
α, β, γ (°)	88.1, 78.8, 62.7	80.8, 80.2, 62.6	80.9, 79.1, 62.7
Resolution (Å)	66.57-2.43	69.75-2.43	64.53-2.25
R_{meas}	0.093 (1.514)	0.103 (1.024)	0.103 (1.131)
$R_{p.i.m}$	0.066 (1.071)	0.073 (0.724)	0.073 (0.8)
$I/\sigma I$	8.2 (1.0)	8.4 (1.4)	7.9 (1.0)
$CC_{1/2}$	0.983 (0.515)	0.995 (0.555)	0.99 (0.962)
Completeness	98.2 (96.9)	98.3 (97.4)	98.2 (96.7)
Multiplicity	2.2 (2.1)	3.5 (3.7)	3.1 (3.1)
**Refinement**			
Resolution	66.66-2.43	69.85-2.43	64.61-2.25
No. of reflections	125031	120841	153878
R_{work}/R_{free}	0.211/0.233	0.224/0.250	0.212/0.227
No. of atoms			
Protein	19414	18863	19824
Ligand	531	404	520
Water	319	233	486
B-factors			
Protein	62.83	61.05	54.39
Ligands	82.15	67.97	59.76
Waters	48.75	48.89	47.12
R.m.s. deviations			
Bond angles (°)	1.377	1.428	1.311
Bond lengths (Å)	0.0046	0.0060	0.0041
