# Supplementary material for: The primary familial brain calcification-associated protein MYORG is an α-galactosidase with restricted substrate specificity
Source: PLoS Biol. 2022 Sep 21;20(9):e3001764. doi: 10.1371/journal.pbio.3001764 (PMC9491548; doi:10.1371/journal.pbio.3001764)
Supplement: S3 Table — Values are the mean of 2 technical repeats ± standard deviations. (PDF) [file pbio.3001764.s009.pdf]

Table S3. Isothermal titration calorimetry results for MYORG. Values are the mean of two technical repeats ± standard deviations.

Compound	K_D (µM)	ΔH (kcal mol^{-1})	ΔG (kcal mol^{-1})	-TΔS (kcal mol^{-1})
DGJ	1.33 ± 0.45	-7.56 ± 0.21	-8.04 ± 0.21	-0.48 ± 0.85
