# Supplementary figures and images for: The primary familial brain calcification-associated protein MYORG is an α-galactosidase with restricted substrate specificity
Source: PLoS Biol. 2022 Sep 21;20(9):e3001764. doi: 10.1371/journal.pbio.3001764 (PMC9491548; doi:10.1371/journal.pbio.3001764)

Original unmodified gel picture taken for Figure S1.

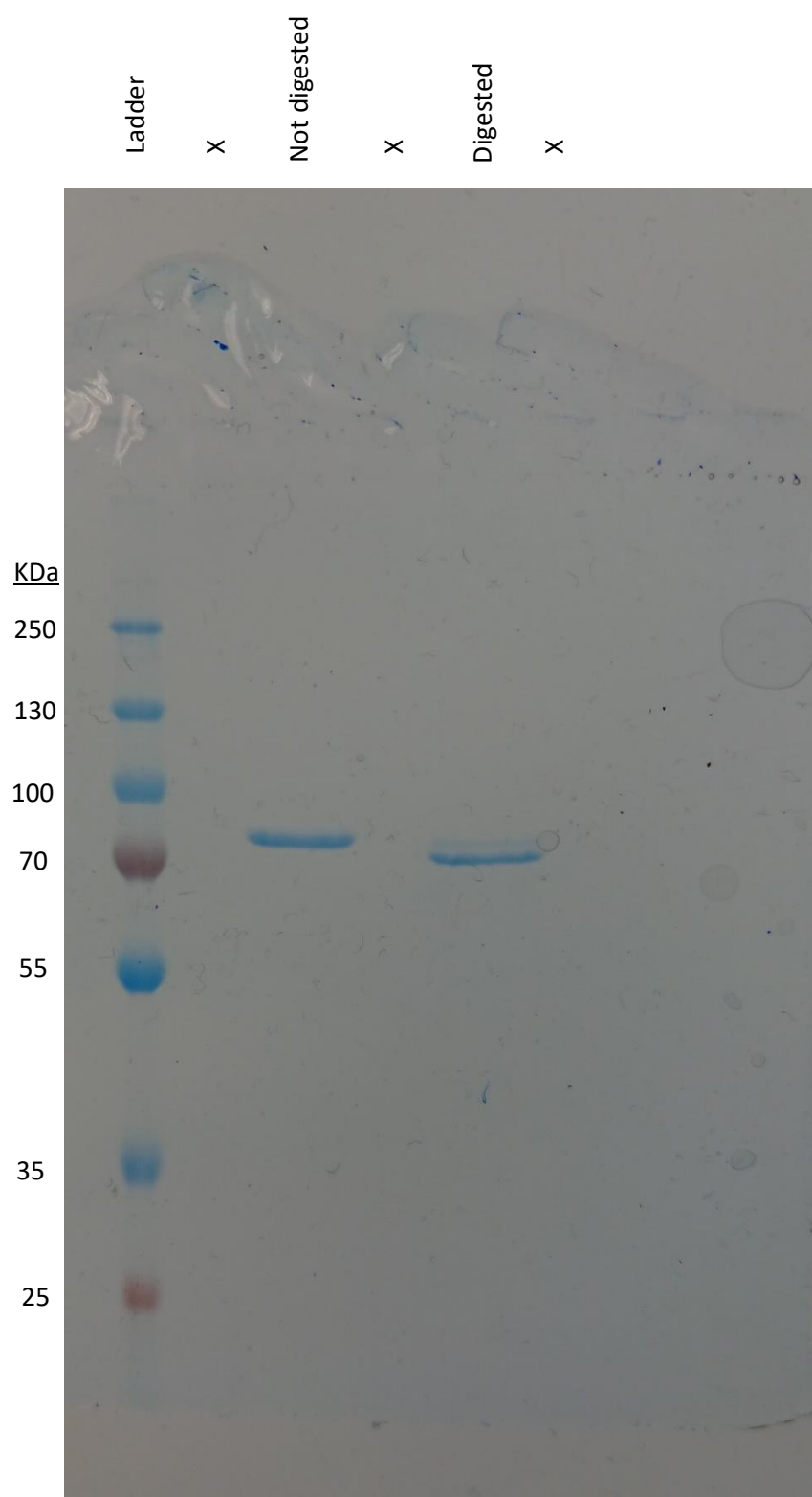

Supplement: S1 Raw Images — (PDF) [file pbio.3001764.s010.pdf]
